# Supplementary material for: A common genetic mechanism underlies morphological diversity in fruits and other plant organs
Source: Nat Commun. 2018 Nov 9;9:4734. doi: 10.1038/s41467-018-07216-8 (PMC6226536; doi:10.1038/s41467-018-07216-8)
Supplement: Supplementary file 1 — Supplementary Information [file 41467_2018_7216_MOESM1_ESM.docx]

**A common genetic mechanism underlies morphological diversity in fruits and other plant organs**

**Wu *et al.***

### 1 Supplementary Methods

2

#### 3

4

1. **Tomato plant materials and construction of the *ovate/sov1/Sltrm5* NILs.** The *ovate*/*sov1* NILs
2. were developed from an initial cross between *Solanum lycopersicum* cv. Yellow Pear and *S.*
3. *pimpinellifolium* accession LA1589. One F_2_ plant (13S116-4) was backcrossed to LA1589 and one
4. BC_1_ plant (13S172-1 wild-type for *fs8.1* and *ovate*, and heterozygous for *sov1*) were crossed to
5. SA29 (carrying *ovate* in the LA1589 background). Selection for *sov1* heterozygosity led to 14S34-
6. 4 that was backcrossed to LA1589 two more times, while maintaining heterozygosity at *ovate* and
7. *sov1*. This line was selfed to yield the 16S88 family that was segregating for both loci. Plant 16S88-
8. 43 is homozygous mutant for both *ovate* and *sov1*. The *Sltrm5-1* allele was created in the LA1589
9. background, backcrossed to LA1589 and then backcrossed to the *ovate/sov1* NIL to remove the
10. CRISPR-Cas9 transgene. This F_1_ was selfed to yield the 16S101 family (*Sltrm5-1*) that was
11. segregating for all three loci. Two hundred F_2_ seedlings (family 16S100) were marker-assisted
12. selected for (1) wild type at all loci; (2) wild type *SlTRM5* and mutant at *ovate/sov1*; (3) mutant
13. *Sltrm5* and wild type at *ovate* and *sov1* loci; and (4) mutant at all three loci. Primers used for
14. genotyping are shown in **Supplementary Data 4**. The *ovate* introgression in the NILs was
15. approximately 73.3kb and included 15 annotated genes 1 whereas that of *sov1* was approximately
16. 3.1Mb and included 183 annotated genes. Multiple screens for recombinants closer to *SlOFP20*
17. were not successful in this cross, suggesting the presence of certain genomic variation that severely
18. limits recombination frequencies.
19. **Potato plant materials.** The potato F_2_ population was developed by self-pollinating a single F_1_
20. hybrid plant. The F_1_ hybrid was generated by crossing DM1-3 as a female to M6. DM1-3 is a
21. doubled monoploid of the cultivated potato *S. tuberosum* Phureja Group 2, 3. The first reference
22. genome sequence for potato was based on DM1-34, 5. M6 is an S7 inbred clone of the wild potato
23. relative *S. chacoense* 6 and was recently sequenced 7. Although diploid potato species are typically
24. self-compatible, M6 is homozygous for a dominant self-incompatibility inhibitor. This gene
25. allowed the F_1_ hybrid to be self-pollinated.
26. For the high-resolution mapping experiment, true seeds were sown from a cross between USW
27. 5337-3 × 77-2102-37, hereafter referred to as C × E8**.** Vigorous seedlings were planted out in boxes

1

1. following a 12 × 8 grid. Leaf tissue was sampled in deep-well microtiter plates for a ‘quick and
2. dirty’ DNA extraction using the NaOH-Tris method. Recombinants were selected from 2500
3. seedlings grown in two batches of 1500 and 1000 seedlings each. The recombinant seedlings were
4. transplanted to 1.1L pots and grown until senescence to harvest tubers. Meanwhile, new leaf tissue
5. of recombinants was re-sampled for high quality DNA extraction using the KingFisher® genomic
6. DNA purification kit (Thermo Scientific, Breda, The Netherlands) according to the manufacturer’s
7. procedures. PCR markers for recombinant analysis in C × E offspring are documented in the
8. Supplementary Data
9. 4, and PCR products were analyzed using the Lightscanner (Idaho Technology Inc., Salt Lake
10. City, UT), followed by high-resolution melting curve analysis.

42

1. **Melon plant materials.** The melon NIL CALC8-1 carrying the fruit shape QTL *fsqs8.1* was
2. developed from a cross between the *indorus* Spanish cultivar “Piel de Sapo” (PS) and the Indian
3. accession PI124112 (CALC) as stated previously9. The advanced-backcross plant 8M42-24,
4. carrying the *fsqs8.1* introgression in the heterozygous state, was backcrossed to PS. Seedlings of
5. the progeny (family 9M7) were screened with markers to select the CALC alleles in the
6. introgression. One plant was found to have a recombinant event within the introgression (9M7-10)
7. that was selfed to generate the progeny test family 13M13. Another plant from this family (9M7-
8. 15), carrying the entire introgression, was selfed and fixed in the subsequent family (10M2). A
9. single plant from this family carrying the homozygous introgression (10M2-30) was selfed and
10. backcrossed to PS to generate the final CALC8-1 NIL and its F_1_ with PS (13M19), respectively.
11. Several 13M19 plants were selfed to obtain sufficient seeds for recombinant screening. Two-
12. hundred sixty seedling of the subsequent family (14M1) were screened with markers (see
13. **Supplementary Data 4**) with a Sequenom MassArray iPLEX at Servicio de Investigaciones
14. Biomédicas, Unidad Central de Investigación (University of Valencia, Valencia, Spain). The
15. genotype of recombinant plants were verified by High Resolution Melting (HRM) or Kompetitive
16. Allele Specific PCR (KASP) markers. Plants with the most informative recombination events 59 (14M1-59, 14M1-56, 14M1-137, 14M1-148, 14M1-223) were selfed to obtain the respective 60 progeny test families 14M34, 14M30, 15M32, 15M33, 14M31.
17. **Cucumber plant materials.** The two cucumber inbred lines WI7238 and WI7239 bear long
18. (FSI>6) and round (FSI ≈1.0) fruits, respectively (**Fig. 6c**). For fine mapping of the *fs2.1* locus, a

2

1. NIL for *fs2.1* (WI7239_*fs2.1*_NIL) was developed with marker-assisted backcrossing using
2. WI7239 as the recurrent parent. The WI7238 × WI7239 F_1_ plant was backcrossed with WI7239 to
3. obtain BC_1_. One plant, *fs2.1*_1C1 that was heterozygous at the *fs2.1* locus was identified by
4. genotyping 96 BC1 progeny with three SSR markers, UW039266 and UW023356 flanking QTL
5. *fs2.1* and the peak SSR marker UW015772. Two more rounds of marker-assisted backcrossing
6. resulted in a heterozygous BC3 plant, *fs2.1*_1H4. Analysis with 100 SSR markers evenly
7. distributed across the seven cucumber chromosomes 10 indicated that the genetic background had
8. returned to the recurrent parent WI7239 except in the *fs2.1* region. This *fs2.1*_1H4 plant was self-
9. pollinated, and homozygous introgression of the *fs2.1* locus was identified from the progeny. This
10. plant, designated as WI7239_*fs2.1*_NIL, was crossed with WI7239 to develop an F_2_ population
11. that is segregating only at the *fs2.1* locus.

74

1. **Fine-mapping of *sov1*.** Initial mapping showed that *sov1* was located between marker 12EP153
2. and 12EP5, a region of ~736 Kb on chromosome 10 11. The families that were selected for
3. additional recombinant screens originated from two separate F_2_ populations (YP × GBL and YP ×
4. T1693) and segregated well in previous generations (**Supplementary Data 4**). The screen using
5. these two flanking markers yielded 17 additional recombinant plants. From each recombinant
6. parent, six homozygous recombinant and six homozygous non-recombinant progeny seedlings
7. were selected and grown in the greenhouse or field at the Ohio State University/OARDC in
8. Wooster, OH, USA for fruit or ovary shape evaluation.
9. **Fine-mapping of melon *fsqs8.1*.** The recombinant family 13M13 was screened with markers
10. between positions 22,310,225 and 28,147,268 on chromosome 8 (**Supplementary Table 4**) and
11. 14 plants homozygous for each PS and CALC alleles were grown in Instituto de Biología
12. Molecular y Celular de Plantas, Valencia Spain, greenhouse facilities. Round shape co-segregated
13. with CALC alleles, demonstrating that *fsqs8.1* was located on the left of Sca86-1149903 marker.
14. Families 14M34, 14M30, 14M31 were progeny tested next year in the same facilities with the
15. same strategy, and allowing to map *fsqs8.1* between markers Sca76-291250 and Sca86-1149903.
16. During the next year, families 15M32 and 15M33 were progeny tested, locating *fsqs8.1* in an
17. interval of 152 kb among markers sca76-197 and sca76-45. Finally, in order to verify the last
18. results, families 16M17 and 16M18, with fixed recombinant events, and the recurrent PS were
19. cultivated in the Agrifood Research and Technology Centre of Aragón, Zaragoza Spain,

3

1. experimental fields. Fruits from both families were round, confirming the results of the previous
2. year.
3. **Fine-mapping of potato *Ro* locus.** Tuber shape of each C × E offspring was visually classified as
4. flat, round, oval and long. The general Mendelian model of segregating marker alleles in this BC1
5. population is ab × bc → ab : ac : bb : bc, where the high resolution melt curves of ab and bc
6. offspring can be identified using the ab and bc parental samples. The curves with the highest
7. melting temperature, due to the absence of SNPs, identifies the bb class. The remaining group of
8. curves should thus be the ac class. Maternal and paternal offspring alleles were deconvoluted from
9. offspring genotypes to create maternal and paternal linkage maps and to pinpoint recombination
10. events from female or male crossover events. Segregation for tuber shape phenotype was
11. compared with marker segregations to identify an interval of at least 0.19 Mb and at most 0.28 Mb
12. between the proximal marker Asp6678 at PGSC4.03 coordinate 48,978,066 and the distal markers
13. Catper20798 and Per20801 at coordinates 49,172,630 and 49,258,672, respectively.
14. **Fine-mapping of cucumber *fs2.1*.** Fine mapping of *fs2.1* was performed using the F_2_ population
15. (NIL-F2) derived from the cross between WI7239_*fs2.1*_NIL and WI7239. WI7239 bears nearly
16. perfect round fruit with an FSI of ~1.0, while WI7239_*fs2.1*_NIL plants set much longer fruit (FSI

111 = 2.9 ± 0.3) (**Fig. 6c**) and their F_1_ plants exhibit intermediate fruit length with FSI close to the mid-

1. parent value of WI7239 and WI7239_*fs2.1*_NIL (2.0 ± 0.2) (**Supplementary Table 6**). For fine
2. mapping, the two flanking SSR markers UW039266 and UW023356 were used to genotype 1536
3. NIL-F_2_ plants, from which 20 recombinants between the two markers were identified. The 1.14
4. Mbp region defined by the two flanking markers was explored for polymorphic indel and dCAPS
5. markers by alignment of resequencing reads of WI7238 and WI7239 12 against the Gy14 cucumber
6. draft genome 13. Five polymorphic Indel markers, one dCAPS marker and the peak SSR marker
7. UW015772 (**Supplementary Table 6**, primer sequences are in **Supplementary Data 4**) were used
8. to genotype the 20 recombinants. The FSI and fruit shape of these plants were consistent with the
9. marker data. All plants carrying WI7238 and WI7239 alleles had long, and round fruits,
10. respectively while all heterozygotes at the *fs2.1* locus had intermediate fruit length and FSI that is
11. similar to F_1_ (**Supplementary Table 6**). Thus, combined with the phenotypic fruit size data, the
12. *fs2.1* locus was delimited by marker fs2.1indel8 and xlsnp10 into a 115.0 kb region on
13. chromosome 2, in which 10 genes were predicted excluding transposons (**Fig. 6c**). The fifth gene,

4

1. *Csa2G227860* which is a homolog of *AtTRM5/SlTRM5*, appears to be the most likely candidate
2. for the cucumber *fs2.1* locus.
3. **RNA expression analyses.** All samples for the gene expression analyses were collected between
4. 10:00 and 10:30 am and preserved immediately. The expression levels of *SlOFP20* in the T_0_
5. *SlOFP20* amiRNA lines were evaluated by semi-quantitative PCR. Total RNA from anthesis
6. flowers was extracted with Trizol (Sigma-Aldrich, St. Louis, MO) and treated with the TURBO
7. DNase (Ambion, Carlsbad, CA). One μg of total RNA were used for first-strand cDNA synthesis
8. using the SuperScript III reverse transcriptase (Invitrogen, Carlsbad, CA). *SlOFP20* and *CAC*
9. (Expósito-Rodríguez et al., 2008) were amplified from the resulting cDNA using *Taq* polymerase
10. (New England Biolabs, Ipswich, MA) with gene-specific primers (**Supplementary Data 4**). The
11. following PCR program was used: 2 minutes at 92 **°**C followed by n cycles of 92 **°**C for 10 seconds,
12. 58 **°**C for 30 seconds and 72 **°**C for 1 minute, n = 18, 24, 26, 28 and 30. The intensities of agarose
13. gel electrophoresis bands were measured by ImageJ software. RNA-seq analyses were performed
14. using anthesis flowers of *ovate* and *sov1* NILs and meristem and young floral buds of LA1589.
15. Total RNA of anthesis flower was extracted as described above. Inflorescences of LA1589 were
16. collected in RNAlater (Sigma-Aldrich, St. Louis, MO) for separation of the meristems and floral
17. buds before RNA extraction. The inflorescence and floral meristems (IM and FM), flower buds at
18. 2 days post floral initiation (dpi), 4 dpi and 6 dpi were dissected using a fine tip tweezer (Dumont,
19. Greenfield, MA) under a dissecting scope. More than 100 meristem or flower buds were included
20. in each replicate. Removal of RNAlater prior to RNA extraction was performed by micropipetting
21. with a drawn out glass Pasteur pipette. Tissues were ground with a pellet pestle. Total RNA was
22. extracted with Trizol (Invitrogen, Carlsbad, CA). The quality of the RNA was assessed using the
23. Agilent Bioanalyzer 2100 and associated RNA Nano 6000 LabChip kit (Agilent, Santa Clara, CA).
24. Only high quality RNA was used to prepare RNA-seq library. Strand-specific RNA-seq libraries
25. with cDNA fragment size of approximately 250 bp were prepared using 1 μg of total RNA
26. following the protocol described previously 14. Barcoded libraries were quantified using a Qubit
27. 2.0 fluorometer (Invitrogen, Carlsbad, CA) and pooled. Libraries were sequenced on the Illumina
28. HiSeq2000 platform to generate single end reads of 51 bp. Sequencing was done at the Genomics
29. Resources Core Facility at Weill Cornell Medical College (New York, NY). Raw reads were
30. processed to remove adapter, polyA/T tails and low quality (quality score < 20) sequences using

5

1. Trimmomatic v0.32 15. Reads were then aligned to the SILVA rRNA database (release 111)
2. (https://[www.arb-silva.de/)](http://www.arb-silva.de/)) to remove rRNA contaminations. Cleaned reads were aligned to the
3. tomato reference genome using tophat2 16 allowing 1 mismatch. Raw counts for each gene were
4. then derived and normalized to reads per kilobase of exon model per million mapped reads
5. (RPKM).

6


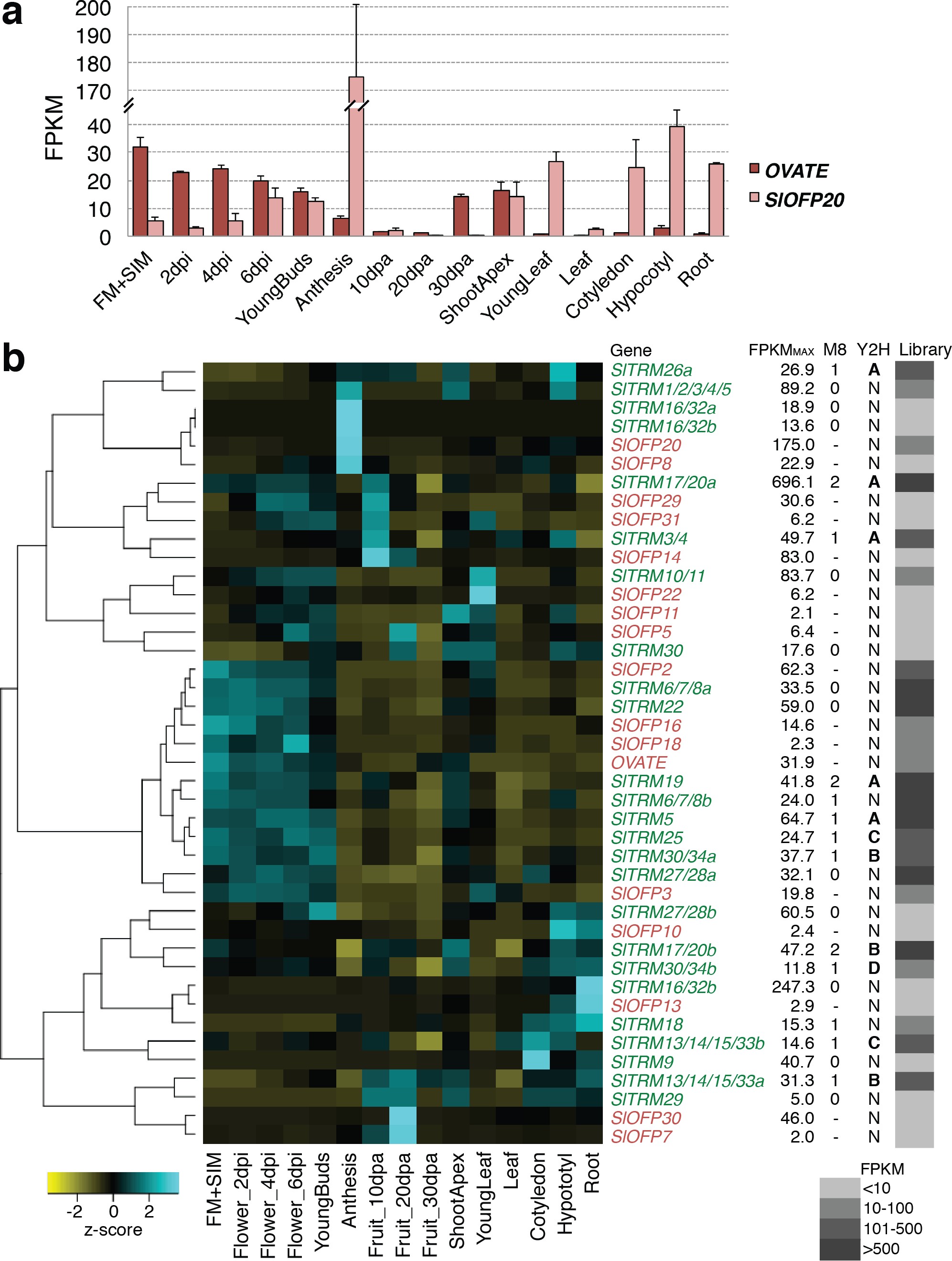


**Supplementary Figure 1. Expression patterns of *OFPs* and *TRMs* in tomato.** (**a**) Expression levels of *OVATE* and *SlOFP20* in various tomato tissues. RPKM, reads per kilobase of transcript per million mapped reads. FM, floral meristem; SIM, sympodial inflorescence meristem; dpi, floral buds collected in number of days post floral initiation; dpa, fruits collected in number of days post anthesis. (**b**) Expression patterns of *SlOFP*s and *SlTRM*s expressed above 2 RPKM in at least one tissue type. RPKMMAX, maximum RPKM of a gene in one of the evaluated tissues. M8, the numbers of M8 motif predicted in SlTRMs. Y2H, the confidence score for the yeast-2-hybrid interactions with A equaling high to D equaling low confidence; N, not applicable since the clone was not identified in the initial OVATE Y2H screen. Library, expression levels of the genes in the Y2H library.


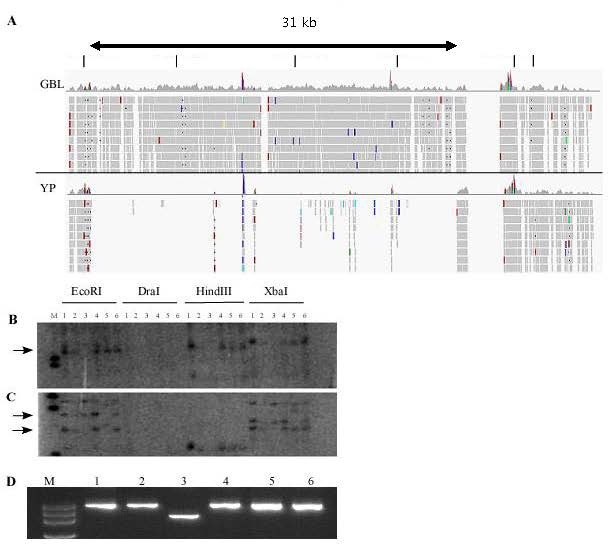


**a**

**b**

**c**

**d**

**Supplementary Figure 2**. **Genomic structure analysis at *sov1***. (**a**) IGViewer showing the deletion in Yellow Pear (YP) compared to Gold Ball Livingston (GBL) between 58,292 kb ~ 58,329 kb of the tomato SL2.40 build. (**b**) Southern blot analysis using *EcoR*I, *Dra*I, *Hind*III, and *Xba*I and a probe G9 (**Supplementary data set 4**). (**c**) Southern blot with probe G4 (**Supplementary data set 4**). (**d**) PCR amplification using 13EP549, the primer set designed from deletion region. Abbreviations: M, Lambda *Hind*III ladder (**b** and **c**), PhiX174 DNA *Hae*III ladder (**d**). Lanes are as follows 1, T1693; 2, Gold Ball Livingston; 3, Yellow Pear; 4, San Marzano; 5, LA1589; 6, Heinz1706. Note the arrow highlighting the missing DNA fragments in the Yellow Pear accession. The fragment size polymorphisms found in LA1589, a wild relative of cultivated tomato, is not associated with change in fruit shape.


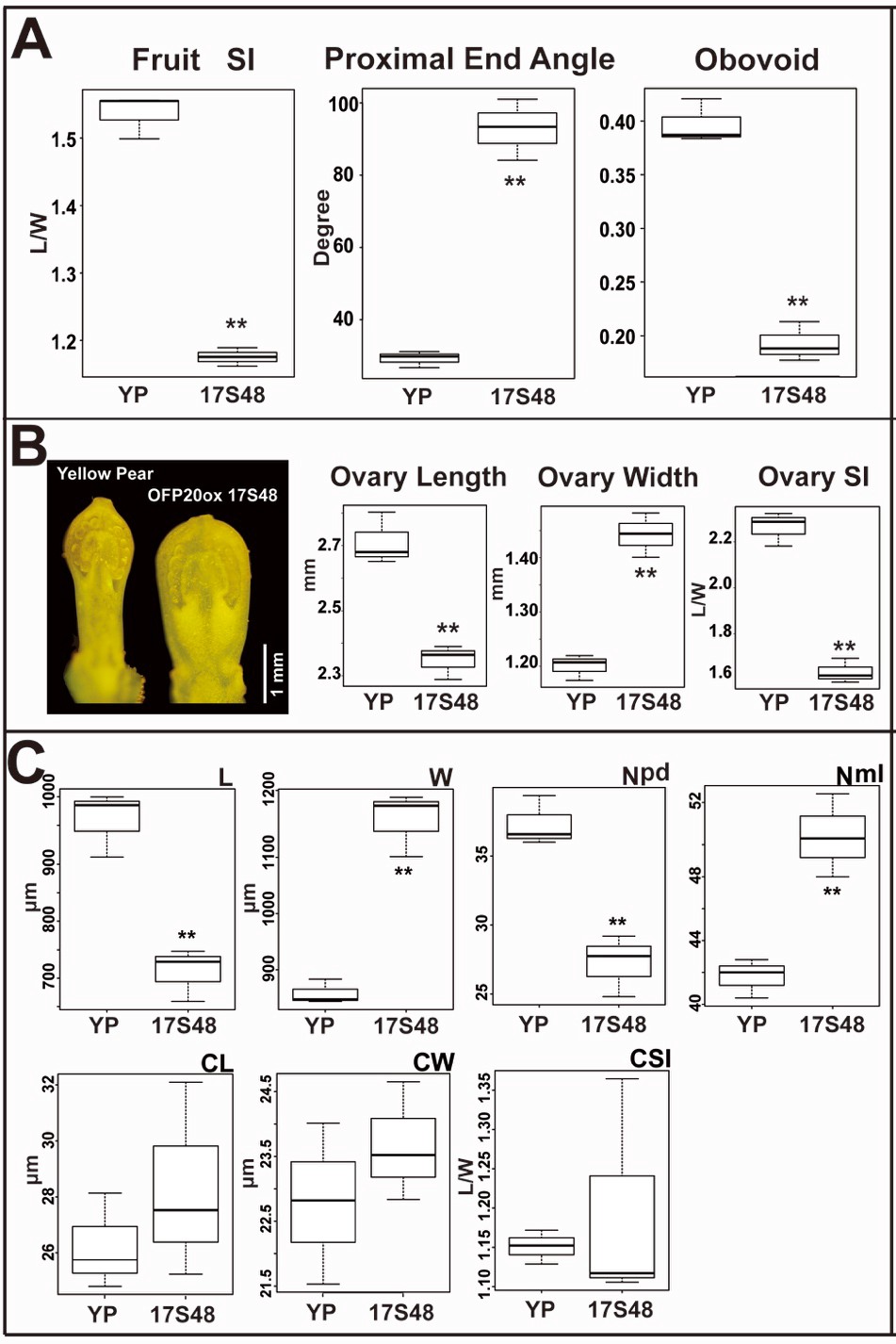


**a**

**b**

**c**

**Supplementary Figure 3. Fruit and ovary morphological traits of *SlOFP20* overexpression lines.** (**a**) Mature fruit shape attributes of the T1 *SlOFP20* over-expressor in the Yellow Pear background, 17S48 derived from T0 142098-4 (the same line as 98-4 shown in Figure 1) compared to wild type Yellow Pear (YP). Fruit SI, fruit shape index; proximal end angle at 15%; obovoid were evaluated with the Tomato Analyzer application 31, 32. (**b**) Anthesis ovaries of Yellow Pear and 17S48. The attributes were evaluated using ImageJ. The scale bar represents 1 mm. (**c**) Cellular attributes at the proximal end of anthesis-stage ovaries of Yellow Pear and family 17S48. Significant differences between transgenic and non-transgenic lines in A-C were determined using the student’s t-Test at: *

<0.05, **, <0.01. L, proximal end area length; W, proximal end area width; N, cell number; pd, proximal-distal; ml, medial-lateral; CL, cell length; CW, cell width; CSI, cell shape index. For each boxplot, the lower and upper bounds of the box indicate the first and third quartiles, respectively. The median is indicated by the center line.


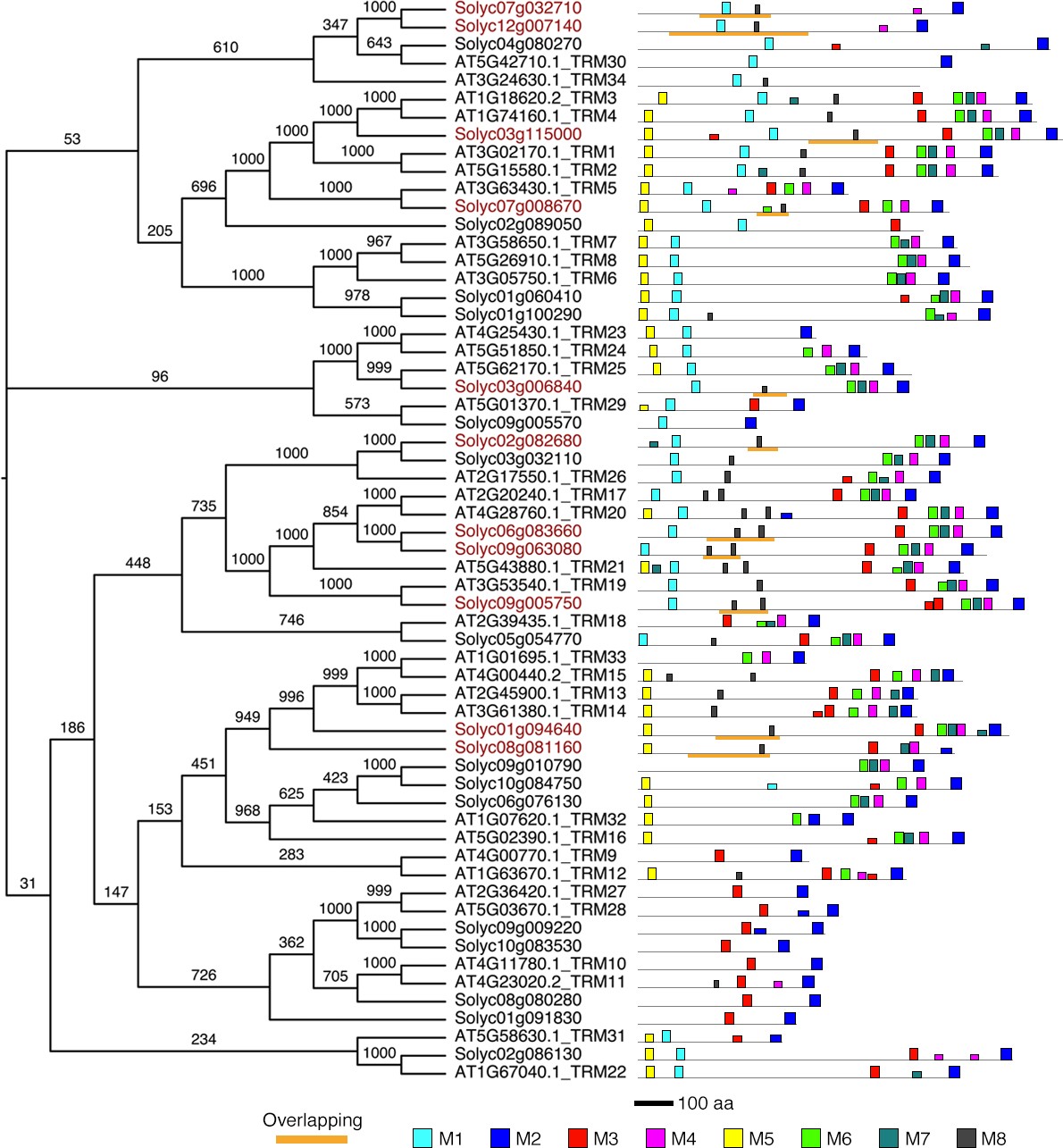


**Supplementary Figure 4. Phylogenetic relationships of SlTRMs and AtTRMs.** The bootstrap values (1000 replicates) are shown on the branches. The 8 motifs identified using MEME are indicated by the color boxes. SlTRMs identified in the OVATE Y2H screen are indicated in red. The overlapping region of independent prey clones corresponding to the same SlTRM is highlighted in orange. The M8 motif in dark grey is expected to interact with OVATE and found in the overlapping regions of all independent prey clones.

## a

**OVATE**

OVATE domain

***** ** * * ** ** *** * ** ** * **** ** * * ****

261 **D**GKVK**E**SFAIVKKSQDPY**ED**FKRSMM**E**MILEKEMFEKN**E**L**E**QLLQCFLSLNGKHYHGVIVEAFS**D**IWETLF


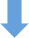

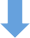

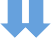

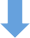

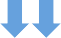

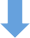


**261V 266**

**V 251**

**279 280**

**V/R V/R**

**265**

**287**

**V/R**

**299 301**

**V V 284 286**

**325**

**A/R**


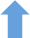

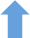

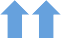


246 TSVTT**D**SFAVVKSSRNPQK**D**FRESMVEMIIENNITTSK**D**L**E**ELLACYLSLNSDEYHDIIIKVFKQIWFEIT

***** ** * * ** ** *** * ** ** * **** ** * * ****

**SlOFP20**

100 aa

OVATE domain

## b

**SlOFP20**

**WT D261V E266V E279V E279R D280V D280R E287V E287R E299V E301V D325A D325R WT E251V D265V D265R E284V E286V**

**OVATE**

**Interaction between OVATE/SlOFP20 and SlTRMs**

9

TRM5 TRM17/20a TRM25

8

7

6

5

4

3

2

1

0

α-galactosidase activity [milliunits / (ml×cell)]

**Supplementary Figure 5. Mapping the domain in SlOFPs that interacts with SlTRMs by *in vitro* mutagenesis.** (**a**) The OVATE domain sequences of OVATE and SlOFP20. Stars denote conserved amino acids between the two OFPs. Arrows denote the charged amino acids that were changed to Valine or Arginine by *in vitro* mutagenesis. (**b**) Y2H interactions of wild-type and mutant versions of OVATE and SlOFP20 with wild-type SlTRM5, SlTRM17/20a and SlTRM25. Bars with an very low activity value (eg. D280V for TRM5 and TRM25) denote a lack of activity in these combinations. Lack of a bar (eg. D261V for TRM25) denotes that the experiment was not conducted.


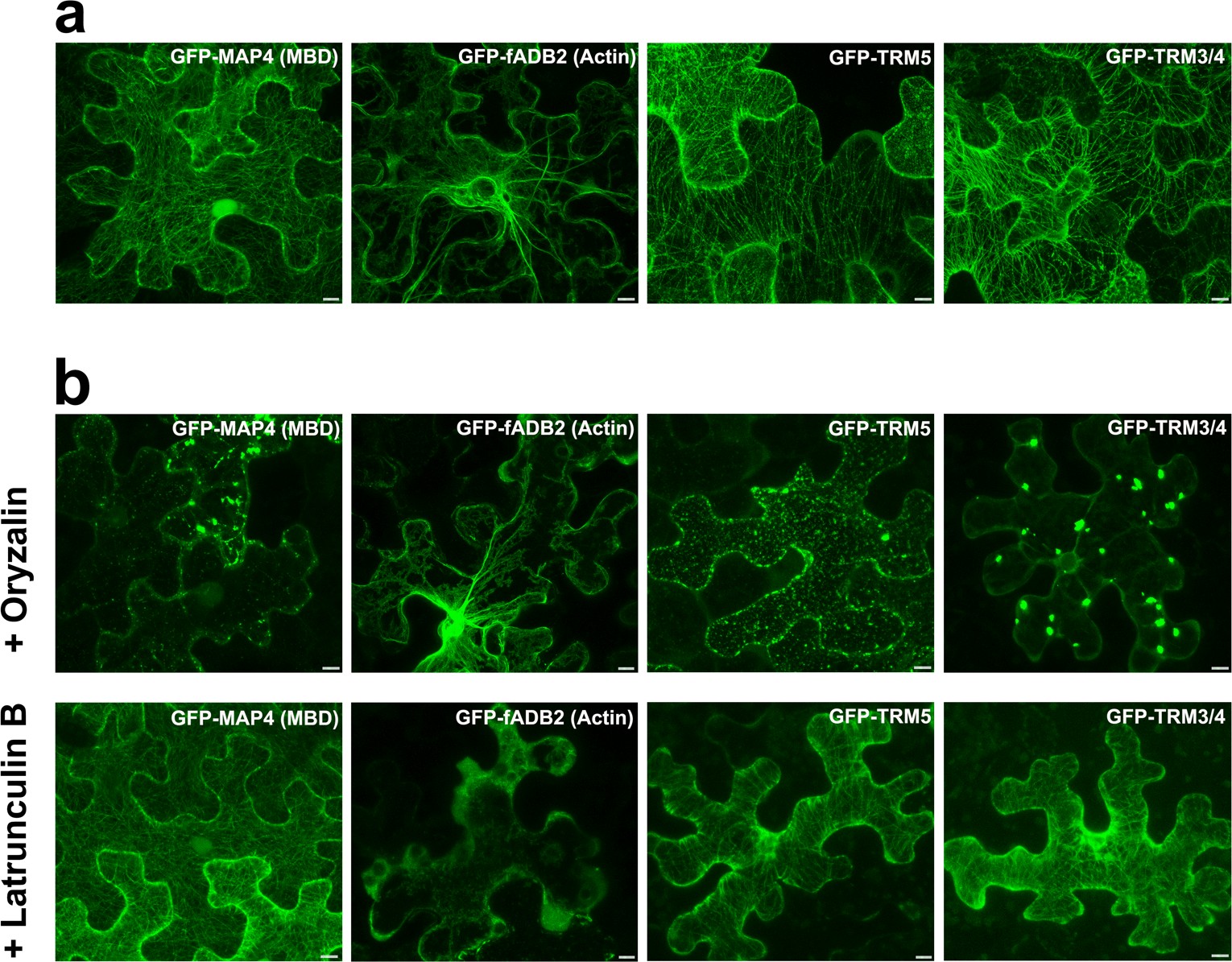


**Supplementary Figure 6. Subcellular localization of SlTRM5, SlTRM3/4, microtubule and actin markers in tobacco *N. benthamiana* cells.** (**a**) Localization patterns of the microtubule marker, GFP-MAP4 43, actin marker, GFP-fADB2 44, GFP-SlTRM5 and GFP-SlTRM3/4. (**b**) Localization patterns of the microtubule and actin markers, GFP-SlTRM5 and GFP-SlTRM3/4 in response to Oryzalin or Lantrunculin B treatments. MAP4 was used as a positive control of depolymerization of the microtubules after the Oryzalin treatment. fADB2 was used as a positive control of depolymerization of the microfilaments after the Latrunculin B treatment. Scale bar 20 µm.


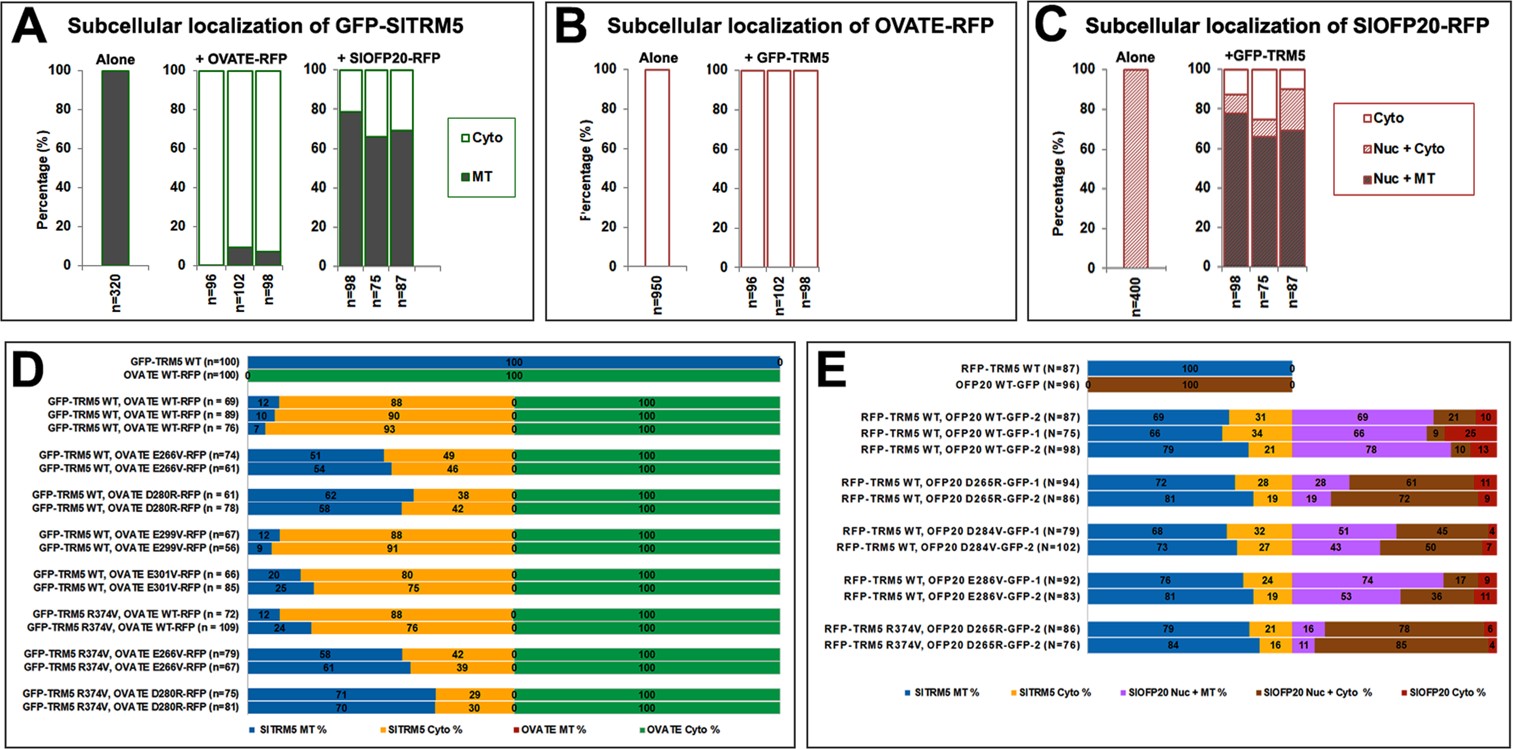


**a**

**b**

**c**

**d**

**e**

**Supplementary Figure 7. Subcellular localization and relocalization of SlTRM5 and SlOFPs when co- expressed.** (**a**) Subcellular localization of SlTRM5 when expressed alone or co-expressed with OVATE or SlOFP20 in three independent experiments. (**b**) Subcellular localization of OVATE when expressed alone or co- expressed with SlTRM5 in three independent experiments. (**c**) Subcellular localization of SlOFP20 when expressed alone or co-expressed with SlTRM5 in three independent experiments. (**d**) Subcellular relocalization of SlTRM5 wild-type/mutant when co-expressed with OVATE wild-type/mutants in two to three independent experiments. (**e**) Subcellular relocalization of SlOFP20 wild-type/mutants and SlTRM5 wild-type/mutant when co- expressed in two to three independent experiments. N, number of cells that were evaluated and expressed both proteins. Cyto, cytoplasm; MT, microtubules; Nuc, nucleus.


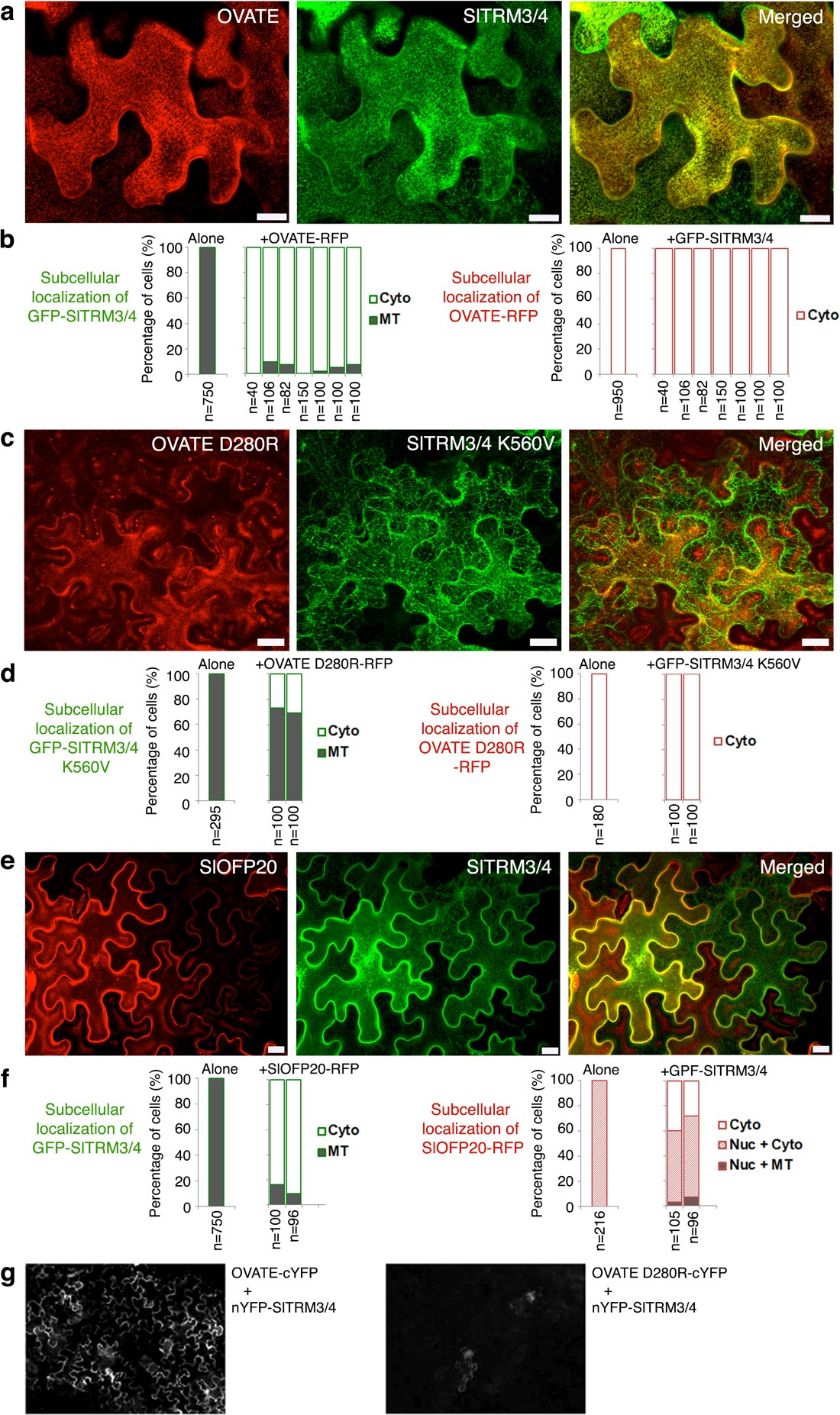


**Supplementary Figure 8. Subcellular localization and relocalization of SlTRM3/4 and SlOFPs when co-expressed.**

(**a**) Co-expression of wild-type OVATE and SlTRM3/4. (**b**) Subcellular localization of SlTRM3/4 or OVATE when expressed alone or co-expressed in seven independent experiments. (**c**) Co-expression of OVATED280R and SlTRM3/4K560V. (**d**) Subcellular localization of SlTRM3/4K560V or OVATED280R when expressed alone or co-expressed in two independent experiments. (**e**) Co-expression of OFP20 and SlTRM3/4. (**f**) Subcellular localization of SlTRM3/4 or SlOFP20 when expressed alone or co-expressed in two independent experiments. Scale bar is 20 µm. (**g**) BiFC with OVATE and SlTRM3/4 or OVATED280R and SlTRM3/4.


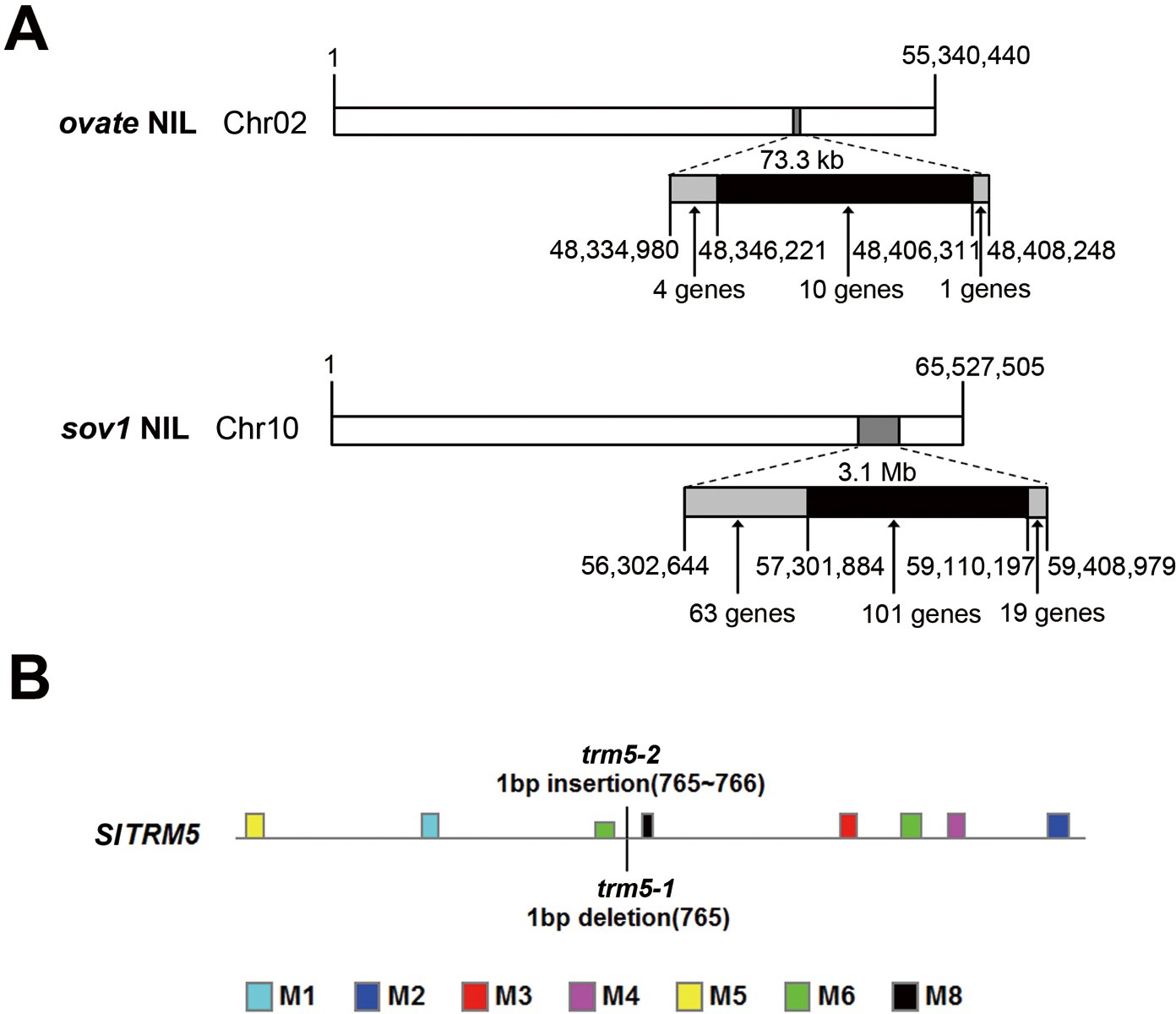


**a**

**b**

**Supplementary Figure 9. Introgression size in the *ovate* and *sov1* NILs and the mutations in two independent *SlTRM5* CRISPR/Cas9 mutant lines.** (**a**) Numbers of genes in the introgressed (black) and crossover (grey) regions based on the tomato ITAG2.4 annotation. (**b**) Positions and types of the mutations in the *SlTRM5* coding sequence are shown based on the nucleotide position starting with the start codon.

# a


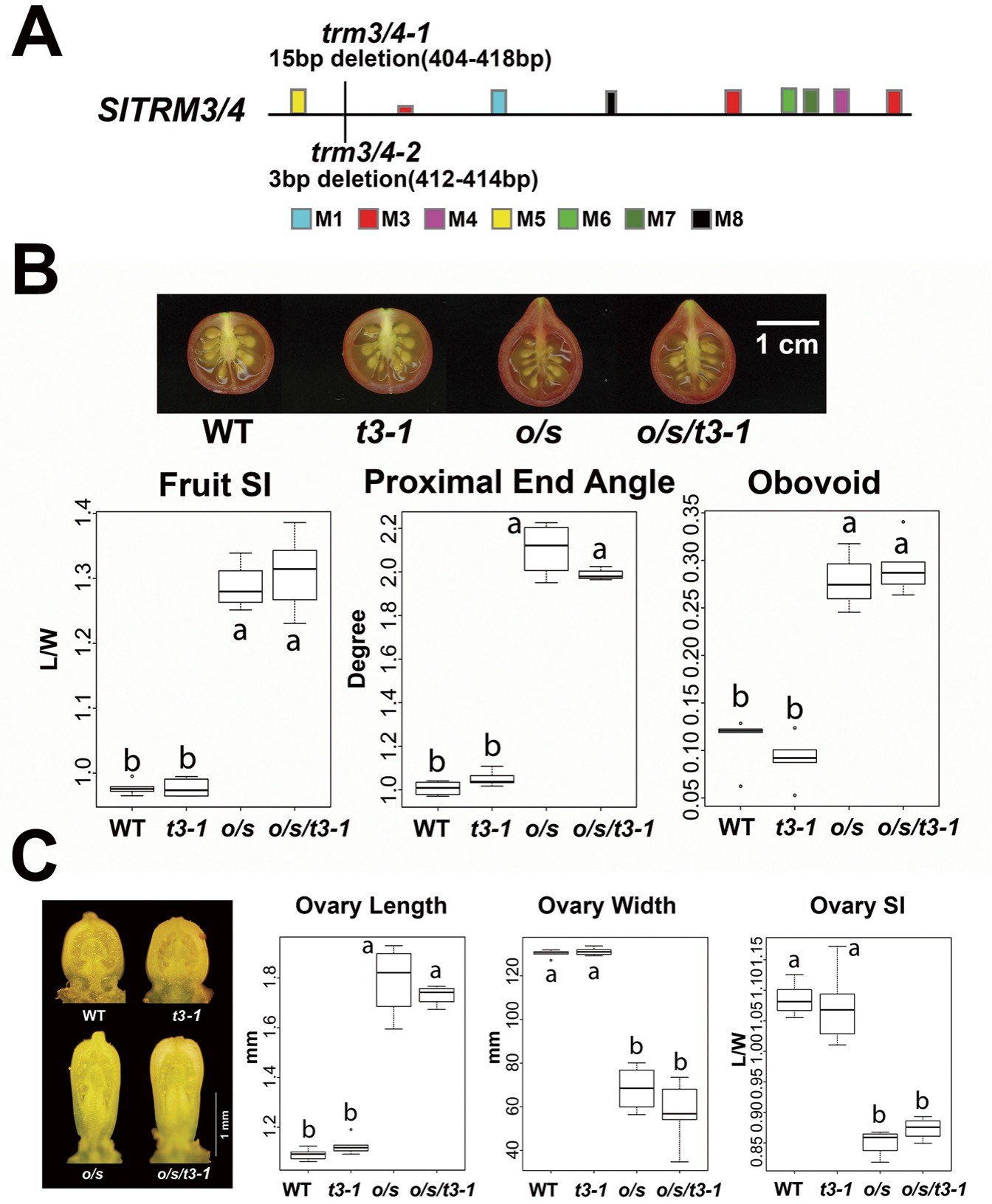


**b**

**c**

**Supplementary Figure 10. Fruit and ovary phenotypes of a *SlTRM3/4* CRISPR/Cas9 mutant line.** (**a**) The mutant alleles and positions of the deletions in the *SlTRM3/4* coding sequence are shown based on the nucleotide position starting with the start codon. (**b**) The effect of the *Sltrm3/4-1* (*t3-1*) allele on mature fruit shape in the wild-type LA1589 and the *ovate*/*sov1* double mutant backgrounds. WT, wild type; *o*, *ovate*; *s*, *sov1*. (**c**) The effect of the *Sltrm3/4-1* (*t3-1*) allele on anthesis-stage ovary shape in the wild-type LA1589 and the *ovate*/*sov1* double mutant backgrounds. The letters in the boxplots indicate the significant differences among different genotypes evaluated by Tukey’s test (α < 0.05). Scale bars for fruit and ovary represent 1 cm and 1 mm, respectively. For each boxplot, the lower and upper bounds of the box indicate the first and third quartiles, respectively. The median and outliers are indicated by the center line and dots, respectively.


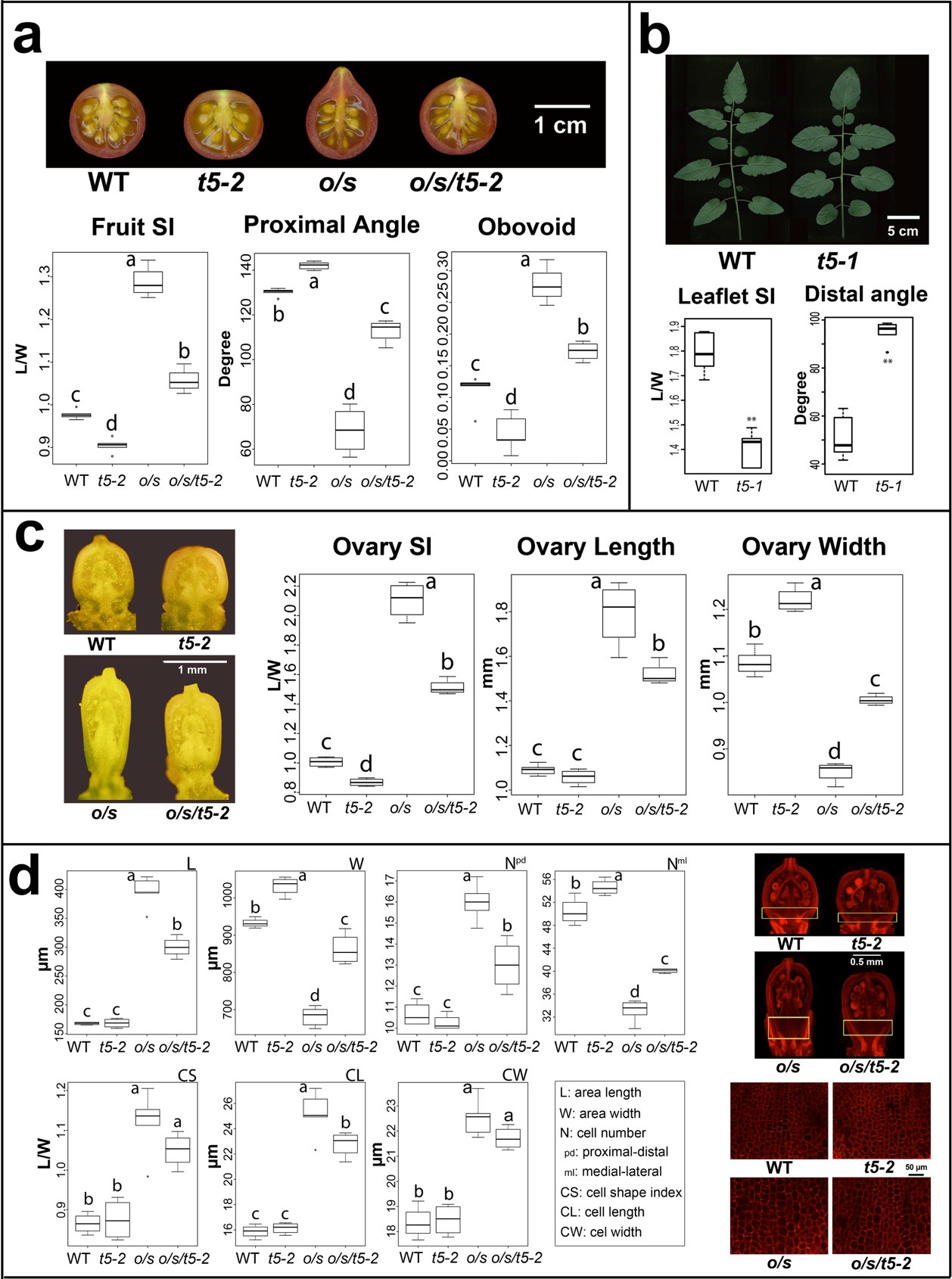


**Supplementary Figure 11. Fruit and ovary phenotypes of a *SlTRM5* CRISPR/Cas9 mutant line (*Sltrm5-2*).** (**a**) The effects of the *Sltrm5-2* (*t5-2*) allele on mature fruit shape in the wild-type LA1589 and the *ovate*/*sov1* double mutant backgrounds. WT, wild type; *o*, *ovate*; *s*, *sov1*. (**b**) The effects of *Sltrm5-1* (*t5-1*) on leaf shape in the LA1589 background.

(**c**) The effects of the *Sltrm5-2* (*t5-2*) allele on anthesis-stage ovary shape in the wild-type LA1589 and the *ovate*/*sov1* double mutant backgrounds. (**d**) Propidium Iodine staining of anthesis-stage ovaries (right top) and proximal area of anthesis ovaries (right bottom) in the four different genotypes. The proximal area of an ovary is indicated by the yellow outline of the box and refers to the length and width traits measured in panels. Histological and cellular evaluations of the proximal end of anthesis ovaries in the different NILs is shown in the middle two panels at the top and the bottom three panels. The letters in the boxplots indicate the significant differences among different genotypes evaluated by Tukey’s test (α < 0.05). For each boxplot, the lower and upper bounds of the box indicate the first and third quartiles, respectively. The median and outliers are indicated by the center line and dots, respectively. The scale bars for fruit, leaf, ovary and cell morphology represent 1 cm, 5cm, 0.5 mm and 50 µm, respectively.


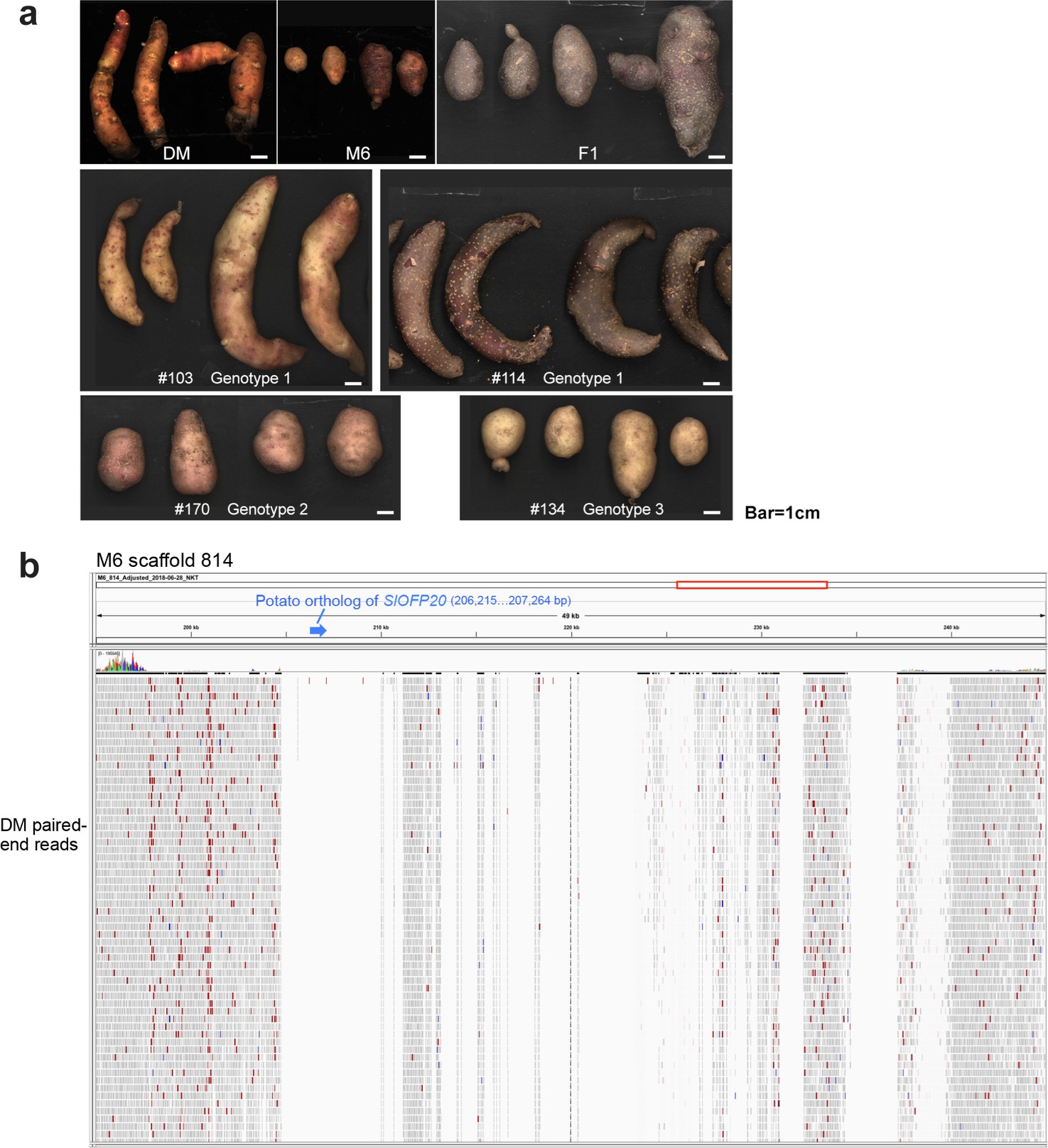


**Supplementary Figure 12. Potato tubers in a segregating population**. (**a**) Potato tubers from the parents, F1 and a segregating F2 population derived from a cross between DM1-3 and M6. The scale bar represents 1 cm. (**b**) IGViewer image aligning DM1-3 reads to the M6 scaffold 814. The gaps denote the deletion in DM1-3 of approximately 30 kb, including *StOFP20* highlighted in blue.

## a


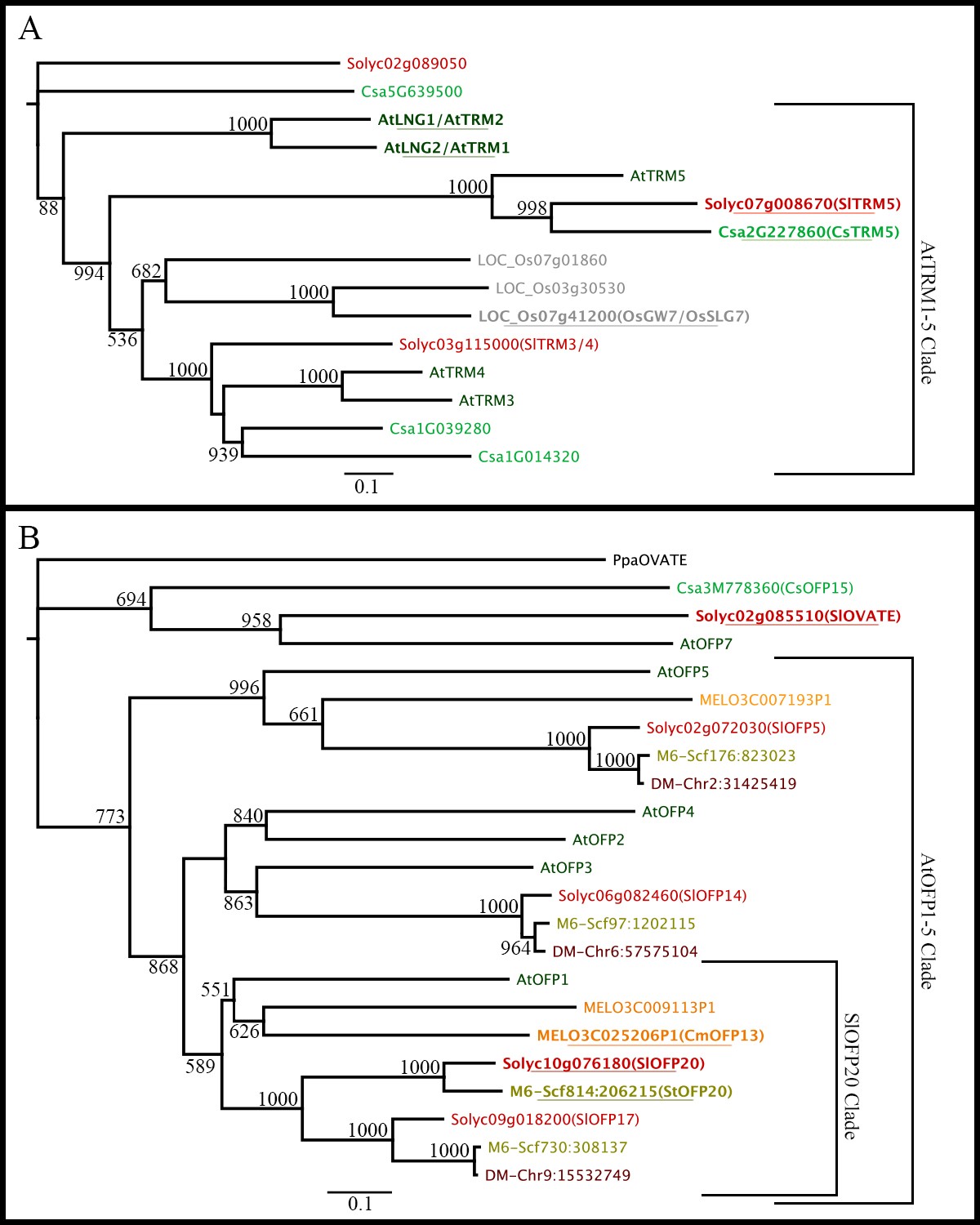


**b**

**Supplementary Figure 13. Phylogeny of the AtTRM1-5 and SlOFP20 clades**. (**a**) AtTRM1-5 clade showing the tomato TRMs in red (Solyc), the Arabidopsis TRMs in dark green (AT), the cucumber TRMs in light green (Csa) and the rice TRMs in grey (LOC). Mutations in TRMs with a discernable phenotype on fruit or grain shape are underlined and bold. (**b**) AtOFP1-5 clade including the SlOFP20 subclade showing the tomato OFPs in red (Solyc or Sl), the Arabidopsis OFPs in dark green (At), the cucumber OFP in light green (Csa), the melon OFPs in orange (MELO) and the potato in dark (DM) or light (M6) brown. Mutations in OFPs with a discernable phenotype on fruit or tuber shape are underlined and bold.

**Supplementary Table 1. Progeny testing of *sov1.***

| Position on chromosome 10  Marker name | | | 57,997,266  12EP153 | 58,067,355  12EP831 | 58,137,322  12EP734 | 58,168,360  12EP834 | 58,182,070  12EP836 | 58,222,158  12EP737 | 58,224,991  13EP427 | 58,293,800  13EP549 | 58,348,731  13EP30 | 58,349,337  13EP37 | 58,368,129  13EP40 | 58,369,797  13EP42 |  |
| --- | --- | --- | --- | --- | --- | --- | --- | --- | --- | --- | --- | --- | --- | --- | --- |
| Grand parent | Parent | Progeny |  | | | | | | | | | | | |  |
| 09S95‐42 | 12S58‐66 | 12S245 | 1 | 2 | 2 | 2 | 2 | 2 | 2 | 2 | 2 | 2 | 2 | 2 |  |
| 09S95‐79 | 12S64‐2 | 13S3 | 0 | 3 | 3 | 3 | 3 | 2 | 2 | 2 | 2 | 2 | 2 | 2 |  |
| 12S50‐17 | 12S198‐2 | 13S27 | 1 | 1 | 1 | 1 | 1 | 2 | 2 | 2 | 2 | 2 | 2 | 2 |  |
| 09S96‐70 | 12S121‐23 | 13S41 | 2 | 2 | 2 | 2 | 2 | 1 | 1 | 1 | 1 | 1 | 1 | 1 |  |
| 12S65‐2 | 12S267‐49 | 13S71 | 1 | 1 | 1 | 1 | 1 | 2 | 2 | 2 | 2 | 2 | 2 | 2 |  |
| 09S95‐17 | 12S48‐20 | 13S1 | 0 | 3 | 3 | 3 | 3 | 3 | 2 | 2 | 2 | 2 | 2 | 2 |  |
|  | 09S95‐50 | 13S2 | 1 | 1 | 1 | 1 | 1 | 1 | 2 | 2 | 2 | 2 | 2 | 2 |  |
| 09S95‐84 | 12S66‐3 | 13S38 | 2 | 2 | 2 | 2 | 2 | 2 | 2 | 2 | 2 | 2 | 2 | 2 |  |
| 09S95‐53 | 12S60‐43 | 12S200 | 2 | 2 | 2 | 2 | 2 | 2 | 2 | 2 | 2 | 2 | 2 | 2 |  |
|  | 09S96‐64 | 13S5 | 3 | 3 | 3 | 3 | 3 | 3 | 3 | 3 | 3 | 3 | 3 | 3 |  |
| 12S91‐2 | 12S268‐158 | 13S72 | 1 | 1 | 2 | 2 | 2 | 2 | 2 | 2 | 2 | 2 | 2 | 2 |  |
| 09S95‐4 | 12S46‐41 | 12S243 | 2 | 2 | 2 | 2 | 2 | 2 | 2 | 2 | 2 | 2 | 2 | 2 |  |
| 09S95‐4 | 12S46‐8 | 12S257 | 2 | 2 | 2 | 2 | 2 | 2 | 2 | 2 | 2 | 2 | 2 | 2 |  |
| 12S91‐2 | 12S268‐172 | 13S73 | 1 | 1 | 2 | 2 | 2 | 2 | 2 | 2 | 2 | 2 | 2 | 2 |  |
| 09S95‐29 | 12S50‐22 | 12S199 | 3 | 3 | 3 | 3 | 3 | 3 | 3 | 3 | 3 | 3 | 3 | 3 |  |
| 09S95‐24 | 12S49‐42 | 12S197 | 3 | 3 | 3 | 3 | 3 | 3 | 3 | 3 | 3 | 3 | 3 | 3 |  |
| 09S95‐53 | 12S194‐2 | 13S25 | 2 | 2 | 2 | 2 | 2 | 2 | 2 | 2 | 2 | 2 | 2 | 2 |  |

Position on chromosome 10

Marker name

chr 11 *sov2* allele 12EP178

Fruit shape progeny test

| Grand parent | Parent | Progeny |  |  |  |  |  |  |  |  |  |  | Average FSI GBL allele (+/‐  sd) | N | Average FSI YP allele (sd) | N | P‐value |
| --- | --- | --- | --- | --- | --- | --- | --- | --- | --- | --- | --- | --- | --- | --- | --- | --- | --- |
| 09S95‐42 | 12S58‐66 | 12S245 | 2 | 2 | 2 | 2 | 2 | 2 | 2 | 2 | 1 | 1 | 1.03 (+/‐0.06) | 6 | 1.17 (+/‐0.07) | 6 | **** |
| 09S95‐79 | 12S64‐2 | 13S3 | 2 | 2 | 2 | 2 | 2 | 2 | 2 | 2 | 2 | 1 | 1.14 (+/‐0.09) | 8 | 1.28 (+/‐0.11) | 8 | *** |
| 12S50‐17 | 12S198‐2 | 13S27 | 2 | 2 | 2 | 2 | 2 | 2 | 2 | 2 | 2 | 3 | 1.28 (+/‐0.08) | 6 | 1.46 (+/‐0.09) | 6 | **** |
| 09S96‐70 | 12S121‐23 | 13S41 | 1 | 1 | 1 | 1 | 1 | 1 | 1 | 1 | 1 | 3 | 1.29 (+/‐0.07 | 5 | 1.37 (+/‐0.10) | 6 | 0.1 |
| 12S65‐2 | 12S267‐49 | 13S71 | 2 | 2 | 2 | 2 | 2 | 2 | 2 | 2 | 2 | 3 | 1.18 (+/‐0.07) | 6 | 1.37 (+/‐0.07) | 6 | **** |
| 09S95‐17 | 12S48‐20 | 13S1 | 2 | 2 | 2 | 2 | 2 | 2 | 2 | 2 | 2 | 2 | 1.15 (+/‐0.07) | 10 | 1.33 (+/‐0.11) | 10 | **** |
|  | 09S95‐50 | 13S2 | 2 | 2 | 2 | 2 | 2 | 2 | 2 | 2 | 2 | 2 | 1.13 (+/‐0.09) | 8 | 1.3 (+/‐0.10) | 8 | *** |
| 09S95‐84 | 12S66‐3 | 13S38 | 2 | 3 | 3 | 3 | 3 | 3 | 3 | 3 | 3 | 1 | 1.11 (+/‐0.09) | 4 | 1.34 (+/‐0.12) | 5 | **** |
| 09S95‐53 | 12S60‐43 | 12S200 | 2 | 2 | 1 | 1 | 1 | 1 | 1 | 1 | 3 | 3 | 1.21 (+/‐0.09) | 6 | 1.36 (+/‐0.05) | 6 | **** |
|  | 09S96‐64 | 13S5 | 3 | 3 | 2 | 2 | 2 | 2 | 2 | 2 | 2 | 2 | 1.27 (+/‐0.08) | 10 | 1.32 (+/‐0.08) | 9 | 0.11 |
| 12S91‐2 | 12S268‐158 | 13S72 | 2 | 2 | 1 | 1 | 1 | 1 | 1 | 1 | 1 | 1 | 1.08 (+/‐0.05) | 5 | 1.17 (+/‐0.09) | 6 | ** |
| 09S95‐4 | 12S46‐41 | 12S243 | 2 | 2 | 2 | 2 | 3 | 3 | 3 | 3 | 3 | 1 | 1.25 (+/‐0.07) | 7 | 1.38 (+/‐0.11) | 6 | **** |
| 09S95‐4 | 12S46‐8 | 12S257 | 2 | 2 | 2 | 2 | 3 | 3 | 3 | 3 | 3 | 1 | 1.08 (+/‐0.07) | 8 | 1.31 (+/‐0.07) | 6 | **** |
| 12S91‐2 | 12S268‐172 | 13S73 | 2 | 2 | 2 | 2 | 1 | 1 | 1 | 1 | 1 | 1 | 1.12 (+/‐0.05) | 6 | 1.27 (+/‐0.07) | 5 | **** |
| 09S95‐29 | 12S50‐22 | 12S199 | 3 | 3 | 3 | 3 | 3 | 3 | 2 | 2 | 2 | 3 | 1.3 (+/‐0.08) | 4 | 1.33 (+/‐0.12) | 6 | 0.5 |
| 09S95‐24 | 12S49‐42 | 12S197 | 3 | 3 | 3 | 3 | 3 | 3 | 3 | 2 | 2 | 1 | 1.14 (+/‐0.05) | 5 | 1.16 (+/‐0.07) | 5 | 0.55 |
| 09S95‐53 | 12S194‐2 | 13S25 | 2 | 2 | 2 | 2 | 2 | 2 | 2 | 2 | 3 | 3 | 1.64 (+/‐0.06) | 6 | 1.97 (+/‐0.10) | 5 | **** |

Marker position coordinates are based on the SL2.40 build.

58,370,526 58,371,858 58,420,478 58,438,602 58,501,989

13EP502 13EP508 13EP512 13EP516

58,508,429

12EP602

58,728,765 58,733,241 59,255,104

12EP846

12EP605

12EP5

11EP176

Abbreviations: FSI, fruit shape index; 0, missing data, 1, homozygous genotype for the GBL allele; 2, heterozygous; 3, homozygous for the YP allele.

P‐value for FSI: * *P* < 0.05; ** *P* < 0.01; ****P* < 0.001; *****P* < 0.0001

*sov1* region recombination breakpoints

### Supplementary Table 2. Clones isolated in the OVATE Y2H screen

| **PBS** | **Tomato gene ID** | **Number of clones** | **Number of**  **independent clones** | **Percentage of total** | **Best hit in Arabidopsis** | **Arabidopsis name** |
| --- | --- | --- | --- | --- | --- | --- |
| A | Solyc07g008670.2.1 | 31 | 13 | 16.8 | AT3G63430 | TRM5 |
| A | Solyc09g005750.2.1 | 27 | 14 | 14.6 | AT3G53540 | TRM19 |
| A | Solyc06g083660.2.1 | 16 | 9 | 8.6 | AT4G28760 | TRM20 |
| A | Solyc03g115000.2.1 | 8 | 5 | 4.3 | AT1G74160 | TRM4 |
| A | Solyc02g082680.2.1 | 8 | 4 | 4.3 | AT2G17550 | TRM26 |
| A | Solyc09g011720.2.1 | 15 | 9 | 8.1 | AT5G01030 |  |
| A | Solyc09g065210.2.1 | 13 | 7 | 7.0 | AT5G19330 | ARIA |
| B | Solyc09g063080.1.1 | 7 | 5 | 3.8 | AT4G28760 | TRM20 |
| B | Solyc01g094640.2.1 | 7 | 3 | 3.8 | AT4G00440 | TRM15 |
| B | Solyc07g032710.2.1 | 5 | 2 | 2.7 | AT3G24630 | TRM34 |
| B | Solyc01g110440.2.1 | 6 | 3 | 3.2 | AT4G34710 | ADC2 |
| C | Solyc03g006840.2.1 | 6 | 2 | 3.2 | AT5G62170 | TRM25 |
| C | Solyc08g081160.2.1 | 2 | 2 | 1.1 | AT2G45900 | TRM13 |
| D | Solyc12g007140.1.1 | 1 | 1 | 0.5 | AT3G24630 | TRM34 |
| D | Solyc03g114410.2.1 | 5 | 1 | 2.7 | AT5G23080 | TOUGH |
| D | Solyc10g080630.1.1 | 4 | 1 | 2.2 | AT3G11960 |  |
| D | Solyc11g008250.1.1 | 3 | 1 | 1.6 | AT3G10420 | SPD1 |
| D | Solyc04g050540.2.1 | 2 | 1 | 1.1 | AT3G04260 | PDE324 |
| D | Solyc01g060040.2.1 | 1 | 1 | 0.5 | AT5G49430 |  |
| D | Solyc01g096250.2.1 | 1 | 1 | 0.5 | AT1G75260 |  |
| D | Solyc02g014740.1.1 | 1 | 1 | 0.5 | AT4G00390 |  |
| D | Solyc02g081250.1.1 | 1 | 1 | 0.5 | AT1G06710 |  |
| D | Solyc05g008780.2.1 | 1 | 1 | 0.5 | AT1G20970 |  |
| D | Solyc05g009080.2.1 | 1 | 1 | 0.5 | AT5G08720 |  |
| D | Solyc05g054930.2.1 | 1 | 1 | 0.5 | AT3G55060 |  |
| D | Solyc07g065370.2.1 | 1 | 1 | 0.5 | AT5G55600 |  |
| D | Solyc11g008080.1.1 | 1 | 1 | 0.5 | AT5G22930 |  |
| D | no match_prey1464246 | 3 | 1 | 1.6 |  |  |
| D | unknown_prey1464243 | 1 | 1 | 0.5 |  |  |
| D | unknown_prey1464245 | 1 | 1 | 0.5 |  |  |
| NA | Solyc09g098040.2.1 | 1 | 1 | 0.5 | AT5G26570 | ATGWD3 |
| NA | Solyc08g062910.2.1 | 1 | 1 | 0.5 | AT1G56070 | LOS1 |
| NA | Solyc03g095250.2.1 | 1 | 1 | 0.5 | AT3G14172 |  |
| NA | Solyc03g096840.2.1 | 1 | 1 | 0.5 | AT3G03150 |  |
| NA | Solyc08g080240.2.1 | 1 | 1 | 0.5 | AT5G18800 |  |
|  | **Total** | **185** |  |  |  |  |

PBS (Predicted Biological Score) is a interaction conficence score. A is the highest confidence rank. NA indicates a clone containing an out-of-frame or antisense sequence.

**Supplementary Table 3. Tomato TRMs and OVATE Y2H screen results**

**Y2H screen using OVATE as bait Prey alignment (aa)**

**Length**

**Number**

**Independent**

**Percentage**

**Maximum covered Overlapping**

**Motif 8^4^**

| **TomatoGeneID AtBestHit^1^ AtName SlName^2^ (aa) PBS^3^ of clones clones of total Start End Start End Num Start Sequence**  Solyc07g008670.2.1 **AT3G63430** AtTRM5 SlTRM5 800 A 31 13 16.76 51 637 304 380 1 367 ESPIVLMRPS | | | | | | | | | | | | | | | |
| --- | --- | --- | --- | --- | --- | --- | --- | --- | --- | --- | --- | --- | --- | --- | --- |
| Solyc09g005750.2.1 | **AT3G53540** | AtTRM19 | SlTRM19 | 990 | A | 27 | 14 | 14.59 | 62 | 853 | 210 | 327 | 242 CSRIAVLKPS  2 | | |
| Solyc06g083660.2.1 | **AT4G28760** | AtTRM20 | SlTRM17/20a | 937 | A | 16 | 9 | 8.65 | 5 | 845 | 176 | 346 | 315 PTRIVVLKPN  249 TKRITVLRPT  2 | | |
|  |  |  |  |  |  |  |  |  |  |  |  |  |  | 311 | PTRIVVLKPS |
| Solyc03g115000.2.1 | **AT1G74160** | AtTRM4 | SlTRM3/4 | 1092 | A | 8 | 5 | 4.32 | 307 | 685 | 436 | 607 | 1 | 553 | ESPIVIMKPA |
| Solyc02g082680.2.1 | **AT2G17550** | AtTRM26 | SlTRM26a | 890 | A | 8 | 4 | 4.32 | 182 | 416 | 280 | 355 | 1 | 305 | PTKIVILRPG |
| 177 TKRITVLRPS  Solyc09g063080.1.1 AT4G28760 AtTRM20 SlTRM17/20b 896 B 7 5 3.78 3 488 167 258 2 | | | | | | | | | | | | | | | |
|  |  |  |  |  |  |  |  |  |  |  |  |  |  | 239 | PTRIVVLKPS |
| Solyc01g094640.2.1 | **AT4G00440** | AtTRM15 | SlTRM13/14/15/33a | 954 | B | 7 | 3 | 3.78 | 40 | 499 | 200 | 358 | 1 | 337 | SSKIVILKPG |
| Solyc07g032710.2.1 | **AT3G24630** | AtTRM34 | SlTRM30/34a | 835 | B | 5 | 2 | 2.70 | 172 | 338 | 185 | 338 | 1 | 301 | SPPIVIMKPL |
| Solyc03g006840.2.1 | **AT5G62170** | AtTRM25 | SlTRM25 | 692 | C | 6 | 2 | 3.24 | 153 | 385 | 297 | 378 | 1 | 320 | YDQVVLLKPK |
| Solyc08g081160.2.1 | AT2G45900 | AtTRM13 | SlTRM13/14/15/33b | 814 | C | 2 | 2 | 1.08 | 36 | 344 | 126 | 332 | 1 | 312 | SNKIVVLKPI |
| Solyc12g007140.1.1 | AT3G24630 | AtTRM34 | SlTRM30/34b | 744 | D | 1 | 1 | 0.54 | 82 | 431 | 82 | 431 | 1 | 298 | APPIVIMRPV |
| Solyc01g100290.2.1 | AT3G58650 | AtTRM7 | SlTRM6/7/8b | 902 |  |  |  |  |  |  |  |  | 1 | 179 | AKPIAVTQPR |
| Solyc03g032110.1.1 | AT2G17550 | AtTRM26 | SlTRM26b | 802 |  |  |  |  |  |  |  |  | 1 | 234 | PAKIVILRPV |
| Solyc05g054770.2.1 | **AT2G39435** | AtTRM18 | SlTRM18 | 656 |  |  |  |  |  |  |  |  | 1 | 188 | STRVIVVKPS |
| Solyc01g060410.2.1 | **AT3G58650** | AtTRM7 | SlTRM6/7/8a | 913 |  |  |  |  |  |  |  |  | 0 |  |  |
| Solyc01g091830.2.1 | **AT4G00770** | AtTRM9 | SlTRM9 | 407 |  |  |  |  |  |  |  |  | 0 |  |  |
| Solyc02g086130.2.1 | **AT1G67040** | AtTRM22 | SlTRM22 | 962 |  |  |  |  |  |  |  |  | 0 |  |  |
| Solyc02g089050.2.1 | AT1G74160 | AtTRM4 | SlTRM1/2/3/4/5 | 734 |  |  |  |  |  |  |  |  | 0 |  |  |
| Solyc04g080270.2.1 | **AT5G42710** | AtTRM30 | SlTRM30 | 1059 |  |  |  |  |  |  |  |  | 0 |  |  |
| Solyc06g076130.2.1 | AT5G02390 | AtTRM16 | SlTRM16/32a | 714 |  |  |  |  |  |  |  |  | 0 |  |  |
| Solyc08g080280.2.1 | **AT4G23020** | AtTRM11 | SlTRM10/11 | 470 |  |  |  |  |  |  |  |  | 0 |  |  |
| Solyc09g005570.2.1 | **AT5G58630** | AtTRM31 | SlTRM31 | 307 |  |  |  |  |  |  |  |  | 0 |  |  |
| Solyc09g009220.2.1 | **AT5G03670** | AtTRM28 | SlTRM27/28 | 482 |  |  |  |  |  |  |  |  | 0 |  |  |
| Solyc09g010790.1.1 | AT5G02390 | AtTRM16 | SlTRM16/32b | 732 |  |  |  |  |  |  |  |  | 0 |  |  |
| Solyc10g083530.1.1 | AT2G36420 | AtTRM27 | SlTRM27/28 | 392 |  |  |  |  |  |  |  |  | 0 |  |  |
| Solyc10g084750.1.1 | **AT5G02390** | AtTRM16 | SlTRM16/32c | 831 |  |  |  |  |  |  |  |  | 0 |  |  |

1Best hits in Arabidopsis were identified by BLAST search against TAIR10 Arabidopsis proteins. Reciprocal best BLAST hits are in bold.

2Tomato genes were named after the most likely orthologs in Arabidopsis determined based on phylogeny.

3PBS, Predicted Biological Score, which is computed to assess the reliability of the interaction. A is the highest confidence rank.

4Motifs were identified using 26 SlTRMs and 34AtTRMs in MEME with following parameters: nmotifs 8, minw 10, maxw 100, minsites 30, maxsites 120.

### Supplementary Table 4. Effects and interactions of *ovate* and *sov1* on fruit and ovary shape attributes

Pr > F

| Population | Traits |  | *ovate* | *sov1* | *ovate×sov1* |
| --- | --- | --- | --- | --- | --- |
| 14S113 | Mature fruit | Shape index Obovoid  Proximal end angle | <0.0001  <0.0001  <0.0001 | <0.0001  0.0062  <0.0001 | <0.0001  0.0146  <0.0001 |
|  | Anthesis ovary shape index |  | <0.0001 | <0.0001 | <0.0001 |
| 15S27 | Mature fruit | Shape index | <0.0001 | 0.0078 | 0.0037 |
|  |  | Obovoid | <0.0001 | 0.1304 | 0.0003 |
|  |  | Proximal end angle | <0.0001 | 0.0022 | 0.0001 |
|  | Anthesis ovary shape index |  | <0.0001 | 0.0022 | 0.0179 |

Significant effects and interactions are shown by the *p* -values computed from the F ratios in ANOVA.

**Supplementary Table 5. Comparisons of fruit and ovary shapes among the *ovate/sov1* partial NILs and *trm5-1* mutant**

|  | | **Fruit shape index^a^** | | | |  |  | **Obovoid^b^** | | |  | **Proximal end angle^c^** | | | |  | **Anthesis ovary shape index^a^** | | |
| --- | --- | --- | --- | --- | --- | --- | --- | --- | --- | --- | --- | --- | --- | --- | --- | --- | --- | --- | --- |
| **Population** | **Genotype^d^** | **mean se** | | | |  | **mean** | **se** | | |  | **mean se** | | | |  | **mean se** | | |
| **14S113** | **33** | 0.933 | ± | 0.005 | c | 0.044 | | ± | 0.010 | c | 160.6 | | ± | 1.29 | a | 1.136 | | ± | 0.020 d |
|  | **32** | 0.921 | ± | 0.004 | c | 0.032 | | ± | 0.007 | c | 164.7 | | ± | 1.01 | a |  | |  |  |
|  | **31** | 0.968 | ± | 0.001 | c | 0.044 | | ± | 0.006 | c | 156.2 | | ± | 0.67 | a | 1.295 | | ± | 0.015 c |
|  | **23** | 0.966 | ± | 0.009 | c | 0.055 | | ± | 0.012 | c | 153.6 | | ± | 2.48 | a |  | |  |  |
|  | **22** | 0.978 | ± | 0.013 | c | 0.062 | | ± | 0.013 | c | 152.1 | | ± | 1.89 | a |  | |  |  |
|  | **21** | 0.979 | ± | 0.018 | c | 0.065 | | ± | 0.015 | c | 151.4 | | ± | 3.21 | a |  | |  |  |
|  | **13** | 1.092 | ± | 0.012 | b | 0.150 | | ± | 0.007 | b | 126.0 | | ± | 1.59 | b | 1.612 | | ± | 0.052 b |
|  | **12** | 1.117 | ± | 0.033 | b | 0.135 | | ± | 0.009 | b | 124.9 | | ± | 7.66 | b |  | |  |  |
|  | **11** | 1.294 | ± | 0.022 | a | 0.214 | | ± | 0.016 | a | 88.5 | | ± | 6.39 | c | 2.223 | | ± | 0.041 a |
| **15S27** | **33** | 0.968 | ± | 0.004 | b | 0.031 | | ± | 0.007 | b | 156.2 | | ± | 1.30 | a | 1.162 | | ± | 0.027 c |
|  | **31** | 0.982 | ± | 0.013 | b | 0.012 | | ± | 0.007 | b | 157.2 | | ± | 3.05 | a | 1.265 | | ± | 0.046 c |
|  | **13** | 1.108 | ± | 0.001 | b | 0.154 | | ± | 0.003 | a | 122.7 | | ± | 1.13 | b | 1.732 | | ± | 0.012 b |
|  | **11** | 1.308 | ± | 0.067 | a | 0.227 | | ± | 0.007 | a | 85.7 | | ± | 4.01 | c | 2.112 | | ± | 0.079 a |
| **16S100** | **WT** | 0.979 | ± | 0.006 | b | 0.049 | | ± | 0.007 | c | 155.3 | | ± | 0.11 | b | 1.037 | | ± | 0.013 c |
|  | ***trm5-1*** | 0.915 | ± | 0.008 | c | 0.004 | | ± | 0.004 | d | 164.9 | | ± | 0.47 | a | 0.895 | | ± | 0.004 d |
|  | ***ovate/sov1*** | 1.255 | ± | 0.012 | a | 0.264 | | ± | 0.005 | a | 72.6 | | ± | 1.73 | d | 1.838 | | ± | 0.036 a |
|  | ***ovate/sov1/trm5-*** | 0.993 | ± | 0.007 | b | 0.134 | | ± | 0.003 | b | 137.8 | | ± | 0.97 | c | 1.330 | | ± | 0.025 b |

aShape index is the ratio of the maximum length over the maximum width of a fruit or ovary.

bObovoid describes the pear-shapedness of a fruit.

cProximal end angles were measured at the position 10% above the proximal end point of a fruit.

dThe first and second numbers in 14S113 and 15S27 represent the genotypes at *ovate* and *sov1* , respectively. 1, homozygous mutant; 3, homozygous WT and 2, heterozygous. Ovary shape indices were measured on homozygous genotypes.

For 14S113, each value represents the mean (± standard error) of 4 plants of the same genotype (8 samples for each plant). For 15S27, each value represents the mean of

2-4 plants of the same genotype (8 samples for each plant). For 16S100, each value represents the mean (± standard error) of 3 plants of the same genotype (8~10

**Supplementary Table 6. Cellular comparisons of anthesis ovaries among the *ovate* /*sov1* NILs and *trm5-1* mutant**

**Genotype**

**Average values of individual plants**

**Mean** ± **SE**

| WT | | 181.52 | 158.96 | 173.63 | 154.38 |  | 167.12 | ± | 6.315 | c |
| --- | --- | --- | --- | --- | --- | --- | --- | --- | --- | --- |
| *sov1* | | 160.40 | 161.18 | 154.80 | 147.82 |  | 156.05 | ± | 3.090 | c |
| *ovate* | | 239.42 | 241.66 | 224.79 | 236.97 |  | 235.71 | ± | 3.764 | b |
| **Proximal area** *ovate* /*sov1* 368.20 433.37 457.13 416.51 418.80 ± 18.812 a | | | | | | | | | | |
| **length^a^ (µm)** | WT | 160.28 | 156.50 | 161.00 | 159.26 | | | ± | 1.396 | c |
|  | *trm5-1* | 139.00 | 149.60 | 154.40 | 147.67 | | | ± | 4.549 | c |
|  | *ovate/sov1* | 331.40 | 298.00 | 341.40 | 323.60 | | | ± | 13.121 | a |
| *ovate/sov1/trm5-1* 255.00 253.60 252.20 253.60 ± 0.808 b | | | | | | | | | | |
| WT | | 933.03 | 937.02 | 933.28 | 906.42 | 927.44 | | ± | 7.064 | a |
| *sov1* | | 925.65 | 921.22 | 862.14 | 865.05 | 893.51 | | ± | 17.307 | a |
| *ovate* | | 786.48 | 811.07 | 790.82 | 810.46 | 799.71 | | ± | 6.448 | b |
| **Proximal area** *ovate/sov1* 771.92 753.37 774.69 729.21 757.30 ± 10.490 c | | | | | | | | | | |
| **width^b^ (µm)** | WT | 891.54 | 823.50 | 871.75 | 862.26 | | | ± | 20.206 | ab |
|  | *trm5-1* | 926.80 | 937.80 | 892.80 | 919.13 | | | ± | 13.544 | a |
|  | *ovate/sov1* | 644.80 | 738.40 | 757.00 | 713.40 | | | ± | 34.718 | c |
| *ovate/sov1/trm5-1* 817.00 871.60 803.00 830.53 ± 20.927 b | | | | | | | | | | |
| WT | | 46.80 | 48.40 | 48.40 | 48.00 | 47.90 | | ± | 0.379 | a |
| *sov1* | | 51.20 | 47.20 | 48.80 | 45.60 | 48.20 | | ± | 1.194 | a |
| **Cell number in** *ovate* | | 38.40 | 38.40 | 38.80 | 41.60 | 39.30 | | ± | 0.772 | b |

**medial-lateral** *ovate/sov1* 37.60 38.00 36.80 33.20 36.40 ± 1.095 c

**direction^c^**

| *trm5-1* | 50.00 | 49.60 | 46.40 | 48.67 | | ± | 1.139 | a |
| --- | --- | --- | --- | --- | --- | --- | --- | --- |
| *ovate/sov1*  *ovate/sov1/trm5-1* | 34.00  39.60 | 35.60  42.80 | 35.20  41.20 | 34.93  41.20 | | ±  ± | 0.481  0.924 | c  b |
| WT | 10.80 | 9.80 | 9.60 | 9.00 | 9.80 | ± | 0.374 | c |
| *sov1* | 9.80 | 9.40 | 9.40 | 8.60 | 9.30 | ± | 0.252 | c |
| **Cell number in** *ovate* | 11.00 | 11.60 | 10.60 | 11.60 | 11.20 | ± | 0.245 | b |

WT 48.80 47.50 47.60 47.97 ± 0.418 a

**proximal-distal** *ovate/sov1* 18.00 17.00 19.20 18.80 18.25 ± 0.486 a

**direction^d^**

| *trm5-1* | 9.40 | 10.00 | 9.40 | 9.60 | | ± | 0.200 | c |
| --- | --- | --- | --- | --- | --- | --- | --- | --- |
| *ovate/sov1*  *ovate/sov1/trm5-1* | 14.60  12.80 | 14.80  11.80 | 15.20  12.00 | 14.87  12.20 | | ±  ± | 0.176  0.306 | a  b |
| WT | 15.78 | 17.46 | 16.90 | 16.14 | 16.57 | ± | 0.378 | d |
| *sov1* | 17.20 | 18.15 | 17.44 | 18.32 | 17.78 | ± | 0.271 | c |
| **Cell length in** *ovate* | 21.38 | 20.04 | 21.35 | 20.37 | 20.79 | ± | 0.341 | b |

WT 10.20 9.00 7.80 9.00 ± 0.693 c

**proximal-distal** *ovate* /*sov1* 22.49 23.28 23.11 23.26 23.04 ± 0.186 a

**direction (µm)^e^**

| *trm5-1* | 15.34 | 14.28 | 17.36 | 15.66 | | ± | 0.902 | c |
| --- | --- | --- | --- | --- | --- | --- | --- | --- |
| *ovate/sov1*  *ovate/sov1/trm5-1* | 24.00  20.14 | 21.52  21.98 | 21.83  19.06 | 22.45  20.39 | | ±  ± | 0.781  0.852 | a  ab |
| WT | 18.70 | 18.33 | 18.23 | 17.91 | 18.29 | ± | 0.163 | c |
| *sov1* | 17.64 | 18.23 | 17.21 | 18.64 | 17.93 | ± | 0.316 | c |
| **Cell width in** *ovate* | 18.84 | 19.46 | 20.09 | 20.58 | 19.74 | ± | 0.378 | b |

WT 15.97 18.73 21.19 18.63 ± 1.510 bc

**medial-lateral** *ovate* /*sov1* 22.00 22.37 21.91 21.57 21.96 ± 0.163 a

**direction^f^ (µm)**

| *trm5-1* | | 17.25 | 17.98 | 18.83 | 18.02 | | ± | 0.457 | a |
| --- | --- | --- | --- | --- | --- | --- | --- | --- | --- |
| *ovate/sov1*  *ovate/sov1/trm5-1* | | 17.72  20.08 | 19.82  21.09 | 23.15  19.09 | 20.23  20.08 | | ±  ± | 1.583  0.578 | a  a |
| WT | | 0.84 | 0.95 | 0.93 | 0.90 | 0.91 | ± | 0.023 | b |
| *sov1* | | 0.97 | 1.00 | 1.01 | 0.98 | 0.99 | ± | 0.008 | a |
| *ovate* | | 1.13 | 1.03 | 1.06 | 0.99 | 1.05 | ± | 0.031 | a |
| **Cell shape** *ovate* /*sov1* 1.02 1.04 1.05 1.08 1.05 ± 0.012 a | | | | | | | | | |
| **index^g^** | WT | 0.92 | 1.10 | 1.14 | 1.05 | | ± | 0.068 | ab |
|  | *trm5-1* | 0.89 | 0.79 | 0.92 | 0.87 | | ± | 0.038 | b |
|  | *ovate/sov1* | 1.35 | 1.09 | 0.94 | 1.13 | | ± | 0.121 | a |
|  | *ovate/sov1/trm5-1* | 1.00 | 1.04 | 1.00 | 1.01 | | ± | 0.014 | ab |

WT 17.44 17.07 18.66 17.72 ± 0.481 a

a,b Area length (a) and width (b) of the proximal area which is the area between the bottom of the locules and that of the whole ovary.

c,d Total cell numbers in the middle of the proximal area in the medial-lateral (c) and proximal-distal (d) directions.

e,f The maximum cell length in the proximal-distal (e) and maximum cell width in the medial-lateral direction (f) of 20 cells

g The ratio of cell length to cell width

Each value represents the mean (± standard error) of 3~4 plants of the same genotype (5 samples for each plant). Means were separated with Duncan's test α <

0.05.

**Supplementary Table 7. Progeny testing of the melon fruit shape locus *fsqs8.1***

| Position on chromosome 8 | 22,310,225 | 22,310,225 | 24,654,693 | 25,001,823 | 25,068,429 | 26,235,312 | 26,329,124 | 26,342,391 | 26,435,530 | 26,454,588 |
| --- | --- | --- | --- | --- | --- | --- | --- | --- | --- | --- |

Marker name Sca10‐7517487 Sca10‐2663375 sca10‐318 Sca76‐1524739 Sca76‐1458133 Sca76‐291250 sca76‐197 sca76‐184 ***CmOFP13*** Sca76‐91032 sca76‐71

| Grand parent | Parent | Progeny |  | | | | | | | | | | |
| --- | --- | --- | --- | --- | --- | --- | --- | --- | --- | --- | --- | --- | --- |
| 8M42‐24 | 9m7‐10 | 13M13 | 2 | 2 | 2 | 2 | 2 | 2 | 2 | 2 |  | 2 | 0 |
| 13M19 | 14M1‐59 | 14M34 | 2 | 2 | 2 | 2 | 2 | 2 | 0 | 0 |  | 3 | 3 |
| 13M19 | 14M1‐56 | 14M30 | 1 | 1 | 1 | 2 | 2 | 2 | 2 | 2 |  | 2 | 2 |
| 13M19 | 14M1‐223 | 14M31 | 2 | 2 | 2 | 2 | 2 | 2 | 2 | 2 |  | 2 | 2 |
| 13M19 | 14M1‐137 | 15M32 | 1 | 1 | 1 | 1 | 1 | 1 | 1 | 2 |  | 2 | 2 |
| 13M19 | 14M1‐148 | 15M33 | 2 | 2 | 2 | 2 | 2 | 2 | 2 | 2 |  | 2 | 2 |
| Position on chromosome 8 26,481,274 | | | | 26,524,440 | 26,531,686 | 26,649,533 | 27,309,283 | 28,147,268 |  |  | | | |
| Marker name sca76‐45 | | | | sca76‐2 | Sca86‐1149903 | sca86‐1032 | sca86‐372 | Sca91‐953336 |  | Fruit shape progeny test | | | |
|  |  |  |  |  |  |  |  |  | Average FSI  PI124112 |  | Average FSI |  |  |
| Grand parent | Parent | Progeny |  |  |  |  |  |  | allele | N | PS allele | N | P‐value |
| 8M42‐24 | 9m7‐10 | 13M13 | 0 | 0 | 1 | 1 | 1 | 1 | 1.09±0.07 | 14 | 1.51±0.10 | 14 | **** |
| 13M19 | 14M1‐59 | 14M34 | 3 | 3 | 3 | 3 | 3 | 3 | 0.99±0.08 | 6 | 0.92±0.07 | 4 | n.s. |
| 13M19 | 14M1‐56 | 14M30 | 2 | 2 | 2 | 2 | 2 | 2 | 0.96±0.06 | 7 | 1.32±0.22 | 6 | ** |
| 13M19 | 14M1‐223 | 14M31 | 2 | 2 | 2 | 1 | 1 | 1 | 1.02±0.11 | 8 | 1.32±0.13 | 8 | **** |
| 13M19 | 14M1‐137 | 15M32 | 2 | 2 | 2 | 2 | 2 | 2 | 0.84±0.06 | 3 | 1.24±0.09 | 3 | ** |
| 13M19 | 14M1‐148 | 15M33 | 1 | 1 | 1 | 1 | 1 | 1 | 0.90±0.05 | 10 | 1.36±0.03 | 2 | **** |

Abreviations: FSI, fruit shape index;0, missing data, 1, homozygous genotype for the PS allele; 2, heterozygous; 3, homozygous for the PI124114 allele.

P‐value for FSI: n.s not significant; * *P* < 0.05; ** *P* < 0.01; ****P* < 0.001; *****P* < 0.0001

*fsqs8.1* region recombination breakpoints

**Supplementary Table 8. Fine-mapping tuber shape QTL *Ro* in the F_1_ offspring of female diploid clone 'C' x male diploid clone 'E'.**

| Coordinates PGSC 4.03  chromosome 10 Marker name | female recombinants male recombinants | |
| --- | --- | --- |
|  | 48,978,066 49,172,630 49,258,672 | 48,978,066 49,172,630 49,258,672 |
|  | Asp6678 Catper20798 Per20801 Asp6678 Catper20798 Per20801 Tuber shape | |
| Plant accession | b b b b b b long b b b b b b long b b b b b b long a a a c c c flat  b b b c c c round b b b c c c flat  a a a b b b Round a a b -- b round | |
| CE2013-527  CE2013-139 CE2013-098 CE2013-168 CE2013-472 CE2013-547 CE2013-555 CE2014-10 CE2014-34 CE2014-74 CE2014-23 CE2014-57 CE2014-36 CE2014-25 CE2013-523 CE2013-110 CE2013-124 CE2013-003 CE2013-119  CE2013-090 |  |  |
|  | b -- a  a -- b  a -- b  a -- b  a -- b | c -- c flat  b -- b long  b -- b round   1. -- b round 2. -- c flat |
|  | b -- b c -- b round  a a a c c c flat  a a a c c c round a a a c c c round a a a c c c flat  a a a c c c flat  a a a c c c round | |

Genotype of the parents: ab x bc and resulting F_1_ genotypes: ab ac bb bc bb = long tuber shape, recessive allele

Ro region

recombination breakpoints

**Supplementary Table 9. Cucumber fruit shape of recombinant F_2_ in the *fs2.1* region**

| Markers  Position on Chinese Long Chromosome 2 | UW039266  10872181 | fs2.1indel13  10958063 | fs2.1indel8  10988274 | *expected fs2.1* | Xl2snp10  11103267 | xl2indel1  11348776 | UW015772  11389000 | xl2indel9  11436317 | xl2indel17  11555556 | UW023356  12013258 | FSI | Fruit Shape |
| --- | --- | --- | --- | --- | --- | --- | --- | --- | --- | --- | --- | --- |

| WI7238 parent 1 | 1 | 1 | 1 | 1 | 1 | 1 | 1 | 1 | 1 | 1 | 6.5 ± 0.4 | Long |
| --- | --- | --- | --- | --- | --- | --- | --- | --- | --- | --- | --- | --- |
| WI7239 parent 2 | 3 | 3 | 3 | 3 | 3 | 3 | 3 | 3 | 3 | 3 | 1.1 ± 0.1 | Round |
| WI7239_fs2.1_NIL x WI7239 F1 | 2 | 2 | 2 | 2 | 2 | 2 | 2 | 2 | 2 | 2 | 2.0 ± 0.2 | Intermediate |
| WI7239_fs2.1_NIL | 1 | 1 | 1 | 1 | 1 | 1 | 1 | 1 | 1 | 1 | 2.9 ± 0.3 | Long |
| 10A2 | 1 | 1 | 1 | 1 | 1 | 1 | 1 | 1 | 1 | 1 | 2.7 ± 0.1 | Long |
| 10A11 | 1 | 1 | 1 | 1 | 1 | 1 | 1 | 1 | 1 | 1 | 2.9 ± 0.3 | Long |
| 9H12 | 2 | 2 | 2 | 2 | 2 | 2 | 2 | 2 | 2 | 2 | 1.7 ± 0.0 | Intermediate |
| 8C9 | 2 | 2 | 2 | 2 | 2 | 2 | 2 | 2 | 2 | 3 | 1.9 ± 0.0 | Intermediate |
| 8D3 | 2 | 2 | 2 | 2 | 2 | 2 | 2 | 2 | 2 | 3 | 2.0 ± 0.1 | Intermediate |
| 2A12 | 2 | 2 | 2 | 2 | 2 | 2 | 2 | 2 | 3 | 3 | 1.9 ± 0.0 | Intermediate |
| 3A9 | 2 | 2 | 2 | 2 | 2 | 2 | 2 | 2 | 3 | 3 | 1.8 ± 0.1 | Intermediate |
| 2D2 | 2 | 2 | 2 | 2 | 2 | 2 | 2 | 3 | 3 | 3 | 1.8 ± 0.0 | Intermediate |
| 9B9 | 2 | 2 | 2 | 2 | 2 | 2 | 2 | 3 | 3 | 3 | 2.0 ± 0.1 | Intermediate |
| 9B5 | 2 | 2 | 2 | 2 | 2 | 2 | 3 | 3 | 3 | 3 | 1.8 ± 0.1 | Intermediate |
| 2G6 | 2 | 2 | 2 | 2 | 2 | 3 | 3 | 3 | 3 | 3 | 1.8 ± 0.1 | Intermediate |
| 5C1 | 2 | 2 | 2 | 2 | 3 | 3 | 3 | 3 | 3 | 3 | 2.3 ± 0.0 | Intermediate |
| 12C1 | 2 | 2 | 2 | 2 | 3 | 3 | 3 | 3 | 3 | 3 | 1.9 ± 0.1 | Intermediate |
| 13A5 | 2 | 2 | 2 | 2 | 3 | 3 | 3 | 3 | 3 | 3 | 1.8 ± 0.0 | Intermediate |
| 14C1 | 2 | 2 | 2 | 2 | 3 | 3 | 3 | 3 | 3 | 3 | 2.1 ± 0.2 | Intermediate |
| 6B2 | 3 | 3 | 2 | 2 | 2 | 2 | 2 | 2 | 2 | 2 | 1.9 ± 0.0 | Intermediate |
| 5D7 | 3 | 3 | 3 | 2 | 2 | 2 | 2 | 2 | 2 | 2 | 1.7 ± 0.1 | Intermediate |
| 9E10 | 3 | 3 | 3 | 3 | 2 | 2 | 2 | 2 | 2 | 2 | 1.2 ± 0.0 | Round |
| 9H7 | 3 | 3 | 3 | 3 | 3 | 2 | 2 | 2 | 2 | 2 | 1.1 ± 0.1 | Round |
| 3C11 | 3 | 3 | 3 | 3 | 3 | 3 | 3 | 3 | 2 | 2 | 1.1 ± 0.1 | Round |
| 8B4 | 3 | 3 | 3 | 3 | 3 | 3 | 3 | 3 | 3 | 2 | 1.0 ± 0.0 | Round |
| 9E12 | 3 | 3 | 3 | 3 | 3 | 3 | 3 | 3 | 3 | 2 | 1.2 ± 0.0 | Round |
| 5B9 | 2 | 3 | 3 | 3 | 3 | 3 | 3 | 3 | 3 | 3 | 1.2 ± 0.0 | Round |
| 9H11 | 3 | 3 | 3 | 3 | 3 | 3 | 3 | 3 | 3 | 3 | 1.2 ± 0.0 | Round |
| 10B10 | 3 | 3 | 3 | 3 | 3 | 3 | 3 | 3 | 3 | 3 | 1.1 ± 0.1 | Round |
| 11C11 | 3 | 3 | 3 | 3 | 3 | 3 | 3 | 3 | 3 | 3 | 1.2 ± 0.1 | Round |

F_2_ was derived from a population derived from a cross between WI7239_fs2.1_NIL and WI7239 Marker position coordinates are based on the Chinese Long (9930 V2.0) build.

Abreviations: FSI, fruit shape index; 1, homozygous genotype for the WI7238 allele; 2, heterozygous; 3, homozygous for the WI7239 allele.

*fs2.1* region recmbinant interval

#### Supplementary References

1. Wu, S. et al. The control of tomato fruit elongation orchestrated by sun, ovate and fs8.1 in a wild relative of tomato. *Plant Sci* **238**, 95-104 (2015).
2. M'Ribu, H.K. & Veilleux, R.E. Effect of genotype, explant, subculture interval and environmental conditions on regeneartion of shoots from in vitro monoploids of a diploid potato species, Solanum phureja Juz. & Buk. *Plant, Cell, Tissue, and Organ Culture* **23**, 171-179 (1990).
3. Paz, M. & Veilleux, R.E. Influence of culture medium and in vitro conditions on shoot regeneration in Solanum phureja monoploids and fertility of regenerated doubled monoploids. *Plant Breeding* **118**, 53-57 (1999).
4. Sharma, S.K. et al. Construction of reference chromosome-scale pseudomolecules for potato: integrating the potato genome with genetic and physical maps. *G3 (Bethesda)* **3**, 2031-2047 (2013).
5. Xu, X. et al. Genome sequence and analysis of the tuber crop potato. *Nature* **475**, 189- 195 (2011).
6. Jansky, S.H., Chung, Y. & Kittipadukal, P. M6: A diploid potato inbred line for use in breeding and genetics research. *J Plant Registrations* **8**, 195-199 (2014).
7. Leisner, C.P. et al. Genome sequence of M6, a diploid inbred clone of the high glycoalkaloid-producing tuber-bearing potato species Solanum chacoense, reveals residual heterozygosity. *Plant J* **94**, 562-570 (2018).
8. Jacobs, J.M. et al. A genetic map of potato (Solanum tuberosum) integrating molecular markers, including transposons, and classical markers. *Theor Appl Genet* **91**, 289-300 (1995).
9. Diaz, A. et al. Mapping and introgression of QTL involved in fruit shape transgressive segregation into 'piel de sapo' melon (cucumis melo l.) [corrected]. *PLoS One* **9**, e104188 (2014).
10. Pan, Y. et al. Round fruit shape in WI7239 cucumber is controlled by two interacting quantitative trait loci with one putatively encoding a tomato SUN homolog. *Theor Appl Genet* **130**, 573-586 (2017).
11. Rodriguez, G.R., Kim, H.J. & van der Knaap, E. Mapping of two suppressors of OVATE (sov) loci in tomato. *Heredity* **111**, 256-264 (2013).
12. Pan, Y., Bo, K., Cheng, Z. & Weng, Y. The loss-of-function GLABROUS 3 mutation in cucumber is due to LTR-retrotransposon insertion in a class IV HD-ZIP transcription factor gene CsGL3 that is epistatic over CsGL1. *BMC Plant Biol* **15**, 302 (2015).
13. Yang, L. et al. Chromosome rearrangements during domestication of cucumber as revealed by high-density genetic mapping and draft genome assembly. *Plant J* **71**, 895- 906 (2012).
14. Zhong, S. et al. High-Throughput Illumina Strand-Specific RNA Sequencing Library Preparation. *Cold Spring Harbor Protocols* **2011**, 940-949 (2011).
15. Bolger, A.M., Lohse, M. & Usadel, B. Trimmomatic: a flexible trimmer for Illumina sequence data. *Bioinformatics* **30**, 2114-20 (2014).
16. Kim, D. et al. TopHat2: accurate alignment of transcriptomes in the presence of insertions, deletions and gene fusions. *Genome Biol* **14**, R36 (2013).

7
