# Supplementary material for: A common genetic mechanism underlies morphological diversity in fruits and other plant organs
Source: Nat Commun. 2018 Nov 9;9:4734. doi: 10.1038/s41467-018-07216-8 (PMC6226536; doi:10.1038/s41467-018-07216-8)
Supplement: Supplementary file 3 — Description of Additional Supplementary Files [file 41467_2018_7216_MOESM3_ESM.pdf]

Description of Additional Supplementary Files

File Name: Supplementary Data 1
Description: Effects of overexpression of SlOFP20 on fruit and ovary shapes in the Yellow Pear background

File Name: Supplementary Data 2
Description: Relative expression, and fruit and ovary shape in the SlOFP20 amiRNA lines

File Name: Supplementary Data 3
Description: Distribution of *ovate* and *sov1* alleles in tomato varieties of different shapes

File name: Supplementary Data 4
Description: Primer list and marker information
