# Supplementary material for: A common genetic mechanism underlies morphological diversity in fruits and other plant organs
Source: Nat Commun. 2018 Nov 9;9:4734. doi: 10.1038/s41467-018-07216-8 (PMC6226536; doi:10.1038/s41467-018-07216-8)
Supplement: Supplementary file 8 — Reporting Summary [file 41467_2018_7216_MOESM8_ESM.pdf]

nature research

Corresponding author(s): Esther van der Knaap

Reporting Summary

Nature Research wishes to improve the reproducibility of the work that we publish. This form provides structure for consistency and transparency in reporting. For further information on Nature Research policies, see Authors & Referees and the Editorial Policy Checklist.

Statistical parameters

When statistical analyses are reported, confirm that the following items are present in the relevant location (e.g. figure legend, table legend, main text, or Methods section).

n/a	Confirmed	
☐	☒	The exact sample size (*n*) for each experimental group/condition, given as a discrete number and unit of measurement
☐	☒	An indication of whether measurements were taken from distinct samples or whether the same sample was measured repeatedly
☐	☒	The statistical test(s) used AND whether they are one- or two-sided<br>*Only common tests should be described solely by name; describe more complex techniques in the Methods section.*
☒	☐	A description of all covariates tested
☒	☐	A description of any assumptions or corrections, such as tests of normality and adjustment for multiple comparisons
☐	☒	A full description of the statistics including central tendency (e.g. means) or other basic estimates (e.g. regression coefficient) AND variation (e.g. standard deviation) or associated estimates of uncertainty (e.g. confidence intervals)
☐	☒	For null hypothesis testing, the test statistic (e.g. *F*, *t*, *r*) with confidence intervals, effect sizes, degrees of freedom and *P* value noted<br>*Give P values as exact values whenever suitable.*
☒	☐	For Bayesian analysis, information on the choice of priors and Markov chain Monte Carlo settings
☒	☐	For hierarchical and complex designs, identification of the appropriate level for tests and full reporting of outcomes
☒	☐	Estimates of effect sizes (e.g. Cohen's *d*, Pearson's *r*), indicating how they were calculated
☐	☒	Clearly defined error bars<br>*State explicitly what error bars represent (e.g. SD, SE, CI)*

Our web collection on statistics for biologists may be useful.

Software and code

Policy information about availability of computer code

Data collection	Collection of fruit shape attributes: Tomato Analyzer v3.0.
Data analysis	Illumina read processing: Trimmomatic v0.32. Illumina read alignment: NovoAlign, BWA-MEM v0.7.13, tophat2. SNP identificaion: SAMtools. Molecular marker development: dCAPS Finder, Primer 3. Read alignment visualization: IGV v2.3.68. Sequence comparison: BLAST. Protein motif analysis: MAST v4.10.1, MEME v4.10.1. Multiple sequence alignment and phylogenetic tree construction: ClustalW, Geneious 10.1.3, FigTree v1.4.2.

For manuscripts utilizing custom algorithms or software that are central to the research but not yet described in published literature, software must be made available to editors/reviewers upon request. We strongly encourage code deposition in a community repository (e.g. GitHub). See the Nature Research guidelines for submitting code & software for further information.

Data

Policy information about availability of data

All manuscripts must include a data availability statement. This statement should provide the following information, where applicable:

- Accession codes, unique identifiers, or web links for publicly available datasets
- A list of figures that have associated raw data
- A description of any restrictions on data availability

Raw sequence reads have been deposited into the National Center for Biotechnology Information sequence read archive (SRA) under accessions SRP090032 [https://trace.ddbj.nig.ac.jp/DRASearch/study?acc=SRP090032]; SRP089970 [https://trace.ddbj.nig.ac.jp/DRASearch/study?acc=SRP089970]; SRA061767 [https://trace.ddbj.nig.ac.jp/DRASearch/submission?acc=SRA061767] for the mRNA seq data; and SRP127270 [https://trace.ddbj.nig.ac.jp/DRASearch/study?acc=SRP127270] for the whole genome sequence data. The normalized expression data are also available at the Tomato Functional Genomics Database (TFGD; http://ted.bti.cornell.edu/) under the experiments D015 and D016.

Field-specific reporting

Please select the best fit for your research. If you are not sure, read the appropriate sections before making your selection.

☒ Life sciences ☐ Behavioural & social sciences ☐ Ecological, evolutionary & environmental sciences

For a reference copy of the document with all sections, see nature.com/authors/policies/ReportingSummary-flat.pdf

Life sciences study design

All studies must disclose on these points even when the disclosure is negative.

Sample size	Sample sizes were chosen based on the testable hypothesis. In general, 4-5 plants and 5-10 fruits or ovaries per plant were evaluated. For the coexpression analyses in N. benthamiana, the number of samples per replicated experiment (2 to 3) ranged nearly always between 80-100.
Data exclusions	We did not exclude data from our experiments.
Replication	Each experiment was at least replicated twice using different plants and different times of the year.
Randomization	Within experiments, plants were randomized such that the same genotype were not grown next to one another on a bench in the greenhouse or the growth chamber.
Blinding	Plants in the greenhouse were coded with a pedigree number that did not indicate the genotype. Thus samples taken from these plants were not selected in an unbiased manner. For the confocal experiments using N. benthamiana, constructs were coded so that the images and cells were not chosen based on expected results. The confocal experiments were repeated many times and by at least 2 different persons in order to attain reproducibility.

Reporting for specific materials, systems and methods

Materials & experimental systems

n/a	Involved in the study
☒	☐ Unique biological materials
☒	☐ Antibodies
☒	☐ Eukaryotic cell lines
☒	☐ Palaeontology
☐	☒ Animals and other organisms
☒	☐ Human research participants

Methods

n/a	Involved in the study
☒	☐ ChIP-seq
☒	☐ Flow cytometry
☒	☐ MRI-based neuroimaging

Animals and other organisms

Policy information about studies involving animals; ARRIVE guidelines recommended for reporting animal research

Laboratory animals	none
Wild animals	none

Field-collected samples | none
